# Whole genome sequence analysis of *Helicobacter pylori* isolates reveals incomplete characterization of antimicrobial resistance mechanisms

**DOI:** 10.1016/j.isci.2025.114077

**Published:** 2025-11-17

**Authors:** Casey Vieni, Tanner Rothstein, Stephen Johnson, Audrey N. Schuetz, Andrew Norgan, Robin Patel

**Affiliations:** 1 Division of Clinical Microbiology, Department of Laboratory Medicine and Pathology, Mayo Clinic, Rochester, MN, USA; 2 Division of Computational Biology, Mayo Clinic, Rochester, MN, USA; 3 Division of Public Health, Infectious Diseases, and Occupational Medicine, Department of Medicine, Mayo Clinic, Rochester, MN, USA

**Keywords:** multi-drug resistant organisms, microbial genomics, clinical microbiology

## Abstract

*Helicobacter pylori*, one of the most common causes of chronic bacterial infections worldwide and a carcinogen, has been associated with significant antimicrobial resistance. Here, whole genome sequencing and phenotypic susceptibility testing to amoxicillin, clarithromycin, levofloxacin, metronidazole, rifampin, and tetracycline of 530 *H. pylori* clinical isolates revealed significant genomic diversity not clustered by geographic location in the United States, and genome-wide association studies revealed incomplete genotypic characterization of antimicrobial resistance mechanisms for amoxicillin, metronidazole, and rifampin. Resistance to amoxicillin, metronidazole, and rifampin was not associated with a particular single-nucleotide polymorphism, the number of mutations in resistant versus susceptible isolates, or a particular mutation hotspot in a gene previously implicated in resistance. This study highlights a need for further elucidation of the genetic basis of antimicrobial resistance (beyond clarithromycin and levofloxacin) in this species.

## Highlights

• *Helicobacter pylori* is genetically diverse without clear geographic clustering across the United States
• Clarithromycin resistance is associated with mutations in the 23S rRNA gene
• Levofloxacin resistance is associated with GyrA mutations
• Amoxicillin, metronidazole, and rifampin resistance is not well genetically characterized

## Graphical abstract

**Figure:**
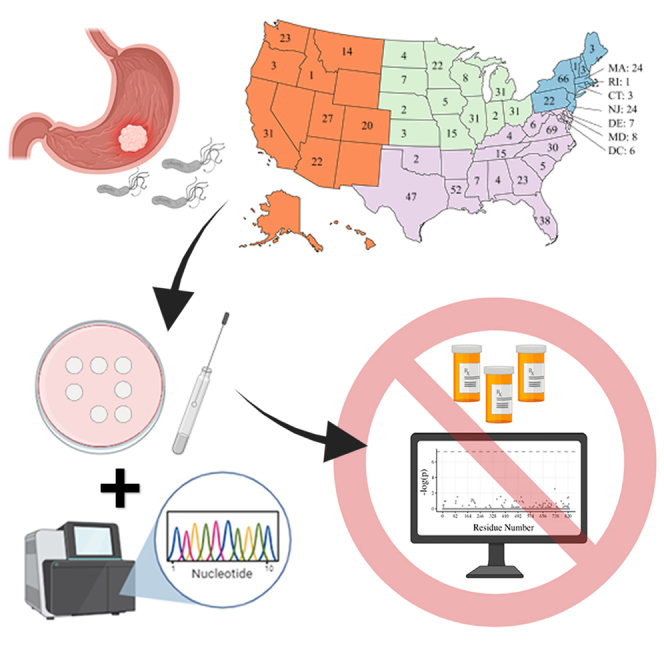

## Introduction

*Helicobacter pylori* is a common cause of chronic bacterial infection worldwide. The bacterium colonizes the gastric mucosa leading to chronic gastritis, with long-term consequences of peptic ulcer disease, adenocarcinoma, and gastric mucosa-associated lymphoid tissue (MALT) lymphoma.^1^^,^^2^^,^^3^ Current guidelines recommend treatment of *H. pylori* positive individuals independent of symptoms.^4^^,^^5^^,^^6^

*H. pylori* treatment has historically been empiric, involving multidrug regimens. First-line treatment has conventionally consisted of amoxicillin, clarithromycin, or metronidazole and a proton pump inhibitor (PPI).^4^^,^^5^^,^^7^ Current first-line regimens for treatment naive patients now includes bismuth quadruple therapy (bismuth, metronidazole, tetracycline, and a PPI) or rifabutin triple therapy (rifabutin, amoxicillin, and a PPI),^6^^,^^8^ although clarithromycin triple therapy is still a widespread first-line therapy.^9^ Other antibiotics included in various combinations for *H. pylori* treatment are amoxicillin, levofloxacin, rifabutin, and/or tetracycline. Unfortunately, antimicrobial resistance in *H. pylori* has become a global challenge.^10^^,^^11^ Antimicrobial susceptibility testing (AST) is routinely performed for other bacterial infections when there is a risk of resistance and has been recommended for *H. pylori* even in primary cases,^4^^,^^12^^,^^13^ as triple therapy guided by AST has better clinical outcomes compared to empiric triple therapy.^13^^,^^14^^,^^15^

Clarithromycin resistance in *H. pylori* is widespread^16^^,^^17^^,^^18^^,^^19^ and is the most characterized resistance mechanism. Resistance to clarithromycin is commonly due to specific single-nucleotide polymorphisms (SNPs) in the 23S ribosomal RNA (rRNA) gene, mostly at A2147G (occasionally reported as A2143G or A2059G depending on the reference strain), followed by A2146G (A2142G) and A2146C (A2142C).^16^^,^^20^^,^^21^^,^^22^ A meta-analysis showed the sensitivity and specificity of the A2146G/C and/or A2147G combination for prediction of clarithromycin resistance in biopsy specimens to be 96% each.^23^

*H. pylori* resistance to the fluoroquinolone levofloxacin has also been well-characterized. Mutations at amino acid residues 87 (N/T to H/I/K/Y) or 91 (D to G/H/M/N/Y/A) of GyrA are most associated with resistance. The N87K and D91N/G/Y mutations combined have a sensitivity and specificity of 97% and 99%, respectively, to genotypically predict fluoroquinolone resistance in *H. pylori* in biopsy specimens.^23^^,^^24^ GyrB mutations may also be associated with lower levels of resistance.^25^^,^^26^^,^^27^^,^^28^^,^^29^^,^^30^^,^^31^^,^^32^

Resistance mechanisms to other antibiotics, including amoxicillin, metronidazole, rifampin, and tetracycline, are less well genetically characterized than mechanisms associated with clarithromycin and levofloxacin resistance. This limits the development and application of genotypic assays, including direct detection in clinical specimens, for amoxicillin, metronidazole, rifampin, and tetracycline resistance, as has been done for clarithromycin resistant *H. pylori*.^21^ Resistance to amoxicillin, rifampin, and tetracycline is uncommon.^18^^,^^33^^,^^34^^,^^35^

When present, amoxicillin resistance has been ascribed to structural changes in penicillin-binding proteins (PBPs), with β-lactamase-mediated resistance being rare.^36^ Mutations in penicillin-binding protein 1 (*pbp1*) have been most strongly associated with amoxicillin resistance. Mutations in *pbp2a* and *fstI* (*pbp3*) and *hofH*, *hopC*, and *hefC* (which affect membrane permeability) have been weakly associated with amoxicillin resistance.^27^^,^^30^^,^^37^

Metronidazole-mediated cell death may occur through an increase in activity of oxygen-radical scavengers, DNA repair enzymes, and/or oxygen tension in the local environment.^27^^,^^38^ While oxygen-insensitive nicotinamide adenine dinucleotide phosphate hydrogen (NADPH) nitroreductase (*rdxA*),^39^^,^^40^^,^^41^^,^^42^^,^^43^ NADPH-flavin nitroreductase (*frxA*), and ferredoxin-like protein (*fdxB*)^44^^,^^45^^,^^46^ may play a role in metronidazole resistance, specific mutations present in resistant isolates are not well-characterized. Several resistant isolates have been shown to have wild-type *rdxA* sequences.^47^^,^^48^^,^^49^ Metronidazole-resistant isolates have been associated with an assortment of missense and frameshift mutations, deletions, and insertions.^47^^,^^48^^,^^50^^,^^51^^,^^52^

Resistance to rifamycins is typically considered to be associated with mutations in the β subunit of RNA polymerase, *rpoB*, but alternative mechanisms may exist. Additionally, phenotypic to genotypic correlation has proven challenging due to the differences between susceptibility to different rifamycins (e.g., between rifampin and rifabutin).^53^^,^^54^^,^^55^^,^^56^ Tetracycline resistance has been associated with mutations in the binding site of the 16S rRNA sequence (particularly positions 926–928) or efflux pump alterations.^18^^,^^30^

To conduct phenotypic AST, an endoscopy is needed to collect specimens for culture, recover an isolate of the microorganism from tissue in culture, and then subject the recovered microorganism to phenotypic AST. This is complicated, as endoscopy is not universally available or otherwise indicated in primary cases, culture is available only in limited settings, and phenotypic AST for *H. pylori* is challenging due to the fastidious nature of the microorganism. Recently, genotypic approaches to resistance detection in *H. pylori* have been described and shown to be amenable to performance on stool or gastric specimens (even formalin-fixed paraffin-embedded tissues). For example, Marrero Rolon et al. described an assay for prediction of clarithromycin resistance (or susceptibility) in *H. pylori* using stool.^21^ There are also commercially available genotypic assays, including the Allplex *H. pylori* & ClariR assay (Seegene), RIDAGENE *Helicobacter pylori* (r-biopharm), Amplidiag *H. pylori* + ClariR (Hologic), BactoReal Kit *H. pylori* ClariRes (ingenetix), and *H. pylori* TaqMan real-time PCR assay (Meridian Bioscience, Inc.) that may be used to identify and test susceptibility of specimens for first-line clarithromycin therapy (i.e., 23S rRNA gene mutations). Genotypic resistance prediction has been reported for other targets, including *rdxA* (metronidazole), the 16S rRNA gene (tetracycline), *pbp1* (amoxicillin), and/or *rpoB* (rifampin) using targeted next-generation sequencing (NGS), such as offered by PyloriDx/PyloriAR (American Molecular Laboratories),^57^ Deeplex HelP (Genoscreen), and *H. pylori* profile (Diagnostics Solutions Laboratories).

Genotypic characterization of phenotypic resistance to antimicrobial agents beyond clarithromycin and levofloxacin remains poorly characterized in *H. pylori*. Consequently, the clinical utility of molecular antimicrobial susceptibility assessment to agents aside from clarithromycin and levofloxacin in *H. pylori* is limited. Here, to better understand genotype-phenotype associations in *H. pylori*, *H. pylori* isolates underwent phenotypic AST and whole genome sequencing (WGS). WGS data were used to create genome-wide associations between phenotypic and genotypic profiles, with a targeted analysis of the genes and SNPs reported to be associated with resistance to amoxicillin, clarithromycin, levofloxacin, metronidazole, and rifampin. Isolates also underwent phenotypic AST to tetracycline; however, there were too few resistant isolates in the collection to perform genome-wide associations between phenotypic and genotypic profiles.

## Results

### Isolate characteristics

**Figure 1:**
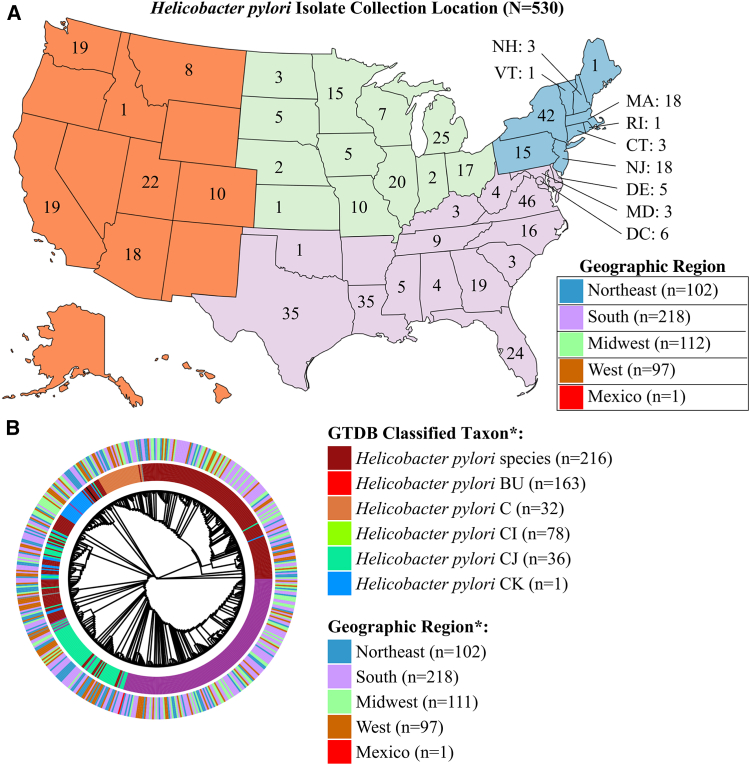
Geographic and genomic clustering of *H. pylori* isolates (A) Geographic origins of *H. pylori* isolates (*N* = 530) submitted for testing to Mayo Clinic from April 2021 to May 2022 colored by geographic region (Northeast, South, Midwest, and West). One isolate originating from Mexico is not shown. (B) Hierarchical clustering of *Helicobacter* isolates at the genus level using the GTDB categorization. The species classification of each isolate is shown according to the GTDB nomenclature with “*H. pylori*” species the type species of the genus. ∗One isolate was excluded from Bactopia’s mashtree workflow because of insufficient coverage.

Of the 530 isolates that passed quality control for WGS (*N* = 530), 50 (9%), 302 (57%), 240, (45%), 374 (71%), 112 (28%), and 7 (1%) were resistant to amoxicillin, clarithromycin, levofloxacin, metronidazole, rifampin, and tetracycline, respectively (Table 1; see Table S1 and Figure S1 for characteristics of all 773 isolates). Isolates’ phenotypic susceptibility patterns did not correlate with failure to pass sequencing QC metrics. Resistance rates were comparable to those in previous reports;^16^^,^^34^^,^^58^ metronidazole resistance was, however, more common (68%) than in a comparably sized dataset (*N* = 544, 30% metronidazole resistant).^44^ Resistance rates for the 773 *H. pylori* isolates tested were comparable to previous reports for levofloxacin (43.5% versus 26.3%–50.4%).^34^ However, resistance rates for clarithromycin (56.9% in this study and 70.4% in our previous study^16^ versus 23.6–40.6% from other studies^34^^,^^58^), amoxicillin (9.7% versus 1.4-5.0%),^34^ and metronidazole (68.4% in this study and 82.4% in our previous study^16^ versus 27.3–58.6% from other studies)^34^ were elevated relative to other reports. Similarly, rifampin resistance rates were higher (27.1%) than previously reported rifabutin resistance rates (0–10.9%,^34^ 1.1%,^59^ and 6.7%^60^). Higher resistance rates observed may reflect a bias for the reference laboratory to receive specimens from treatment-resistant cases. 342 isolates were resistant to two antibiotics, with 132, 126, 66, 17, and 1 resistant to 2, 3, 4, 5, or all 6 antibiotics tested, respectively. Resistance to both clarithromycin and metronidazole was most common, with 251 isolates meeting that categorization, followed by 186 isolates resistant to both clarithromycin and levofloxacin (Table S2). The isolates were approximately equally distributed from across the United States with 102, 218, 112, and 97 isolates, respectively, from the Northeast, South, Midwest, and West, and one isolate from Mexico (Figure 1A).

**Table 1:** Results of AST of analyzed *Helicobacter pylori* isolates by reference agar dilution method

Antibiotic	MIC (μg/mL) breakpoint	Interpretive category^a^	Isolates, *N* = 530 (%)
Amoxicillin	≤0.12	S	480 (90.6)
	>0.12	R	50 (9.4)
Clarithromycin	≤0.25	S	226 (42.6)
	0.5	I	2 (0.4)
	>0.5	R	302 (57.0)
Levofloxacin	≤1	S	290 (54.7)
	>1	R	240 (45.3)
Metronidazole	≤8	S	156 (29.4)
	>8	R	374 (70.6)
Rifampin	≤1	S	384 (72.5)
	>1	R	146 (27.5)
Tetracycline	≤1	S	523 (98.7)
	>1	R	7 (1.3)

MIC, minimum inhibitory concentration; S, susceptible; I, intermediate; R, resistant.

a Clarithromycin MICs were interpreted using CLSI breakpoints. Amoxicillin, levofloxacin, metronidazole, rifampin, and tetracycline MICs were interpreted using EUCAST breakpoints.

The average number of variants called in each isolate was 64,985 (median: 63,933; range: 9,827–78,796) or 3.90% (range: 0.59%–4.72%) of the *H. pylori* 26695 genome. Considering the genetic diversity of *H. pylori*,^61^ a hierarchical cluster analysis at the genus level according to the Genome Taxonomy Database (GTDB) taxonomy category was performed to assess for differences between *H. pylori* isolates (Figure 1B).^62^ All isolates were defined as *Helicobacter* at the genus level. However, diversity was observed at lower taxonomic levels, with 223 isolates classified as “*Helicobacter pylori* species,” and 170 classified as *H. pylori* BU. As of the time of writing, GTDB species representatives correspond to isolate HE147/09 (GCF_900120335.1) for *H. pylori* BU (*n* = 163); an isolate (GCF_000277405.1) from the gastric antrum of an Amerindian resident from the Shimaa village of Peru for *H. pylori* C (*n* = 32); an isolate (GCA_000448465.1) from a gastric specimen obtained in Tuquerres, Colombia, for *H. pylori* CI (*n* = 78); an isolate (GCF_003637685.1) from Perth, Australia, for *H. pylori* CJ (*n* = 36); and an isolate (GCA_000448465.1) from Perth, Australia, for *H. pylori* CK (*n* = 1), highlighting genetic diversity among this collection’s isolates. *H. pylori* isolates did not cluster according to geographic region (Figure 1B).

### GWASs reveal correlational differences for phenotypic resistance among tested antibiotics

Genome-wide association studies (GWASs) showed several unexpected genetic correlations with phenotypic resistance (Table 2).

**Table 2:** SNPs from a genome wide association study in *H. pylori* ATCC 26695 correlated with phenotypic resistance to amoxicillin, clarithromycin, levofloxacin, metronidazole, and rifampin through a Fisher exact test (*p* ≤ 5e-8)

Resistance phenotype	Gene	Gene description^b^	Nucleotide index	-log(*p*) (*p* ≤ 5e-8)	Number of susceptible isolates	Number of resistant isolates	Amino acid index	-log(p) (*p* ≤ 5e-8)
Amoxicillin (S: 480; R: 50)	*HP_0089*	pfs protein (pfs)	95553	7.51	1	8	120	NA
	*HP_0090*	malonyl coenzyme A-acyl carrier protein transacylase (fabD)	96023	7.35	1	7	268	7.35
	*HP_0137*	hypothetical protein; identified by GeneMark putative	147545	7.51	1	8	124	NA
	*HP_0215*	CDP-diglyceride synthetase (cdsA)	223935	7.85	2	9	15	NA
	*HP_0223*	ATP-dependent protease (sms)	231050	7.35	0	7	26	NA
	*HP_0522*	cag pathogenicity island protein (cag3)	549180	7.51	0	7	349	NA
	*HP_0538*	cag pathogenicity island protein (cag17)	570351	7.51	1	8	161	7.51
			570352	7.51	1	8		
			570353	7.51	1	8		
	Intergenic region between HP_1185 & HP_1186		1255570	7.51	1	8	NA	NA
	*HP_1218*	glycinamide ribonucleotide synthetase (purD)	1294936	7.35	0	7	274	NA
	*HP_1253*	tryptophanyl-tRNA synthetase (trpS)	1329463	7.35	0	7	155	7.35
	*HP_1347*	uracil-DNA glycosylase (ung)	1407354	7.51	0	8	176	8.44
Clarithromycin (S: 226; R: 304^a^)	*HP_r01*	23S rRNA	447394	12.58	1	54	NA	NA
			447395	69.46	1	206	NA	NA
	*HP_0537*	cag pathogenicity island protein (cag16)	568906	7.37	116	82	62	7.37
	*HP_0547*	cag pathogenicity island protein (cag26)	580288	8.50	118	80	123	8.50
			580319	7.38	123	91	133	7.38
			581324	8.14	114	77	468	8.38
			582441	7.79	117	82	841	8.01
			583037	7.86	132	100	1039	8.10
	*HP_0887*	vacuolating cytotoxin	938442	7.04	128	101	10	7.47
			938467	7.92	130	97	18	8.38
			938473	8.75	137	102	20	9.06
			938484	7.36	103	68	24	7.63
			938486	8.26	140	108		
			938490	7.69	128	96	26	9.50
			938491	9.00	138	102		
			938494	9.00	138	102	27	9.50
			940644	7.38	134	105	744	7.37
			940645	7.38	134	105		
			940652	8.02	117	81	746	NA
			941193	7.52	103	207	927	7.52
			941205	7.37	105	209	931	7.37
Levofloxacin (S: 290; R: 374)	*HP_r01*	23S rRNA	447395	10.03	77	130	NA	NA
	*HP_0701*	DNA gyrase, sub A (gyrA)	785771	21.89	49	91	87	55.83
			752782	17.85	1	52	91	22.83
Metronidazole (S: 156; R: 374)	*HP_r01*	23S rRNA	447395	8.51	51	156	NA	NA
Rifampin (S: 563; R: 209)	*p* ≤ 5e-8 not observed for any SNPs enriched in rifampin-resistant isolates							

S, susceptible; R, resistant.

Rifampin resistance was not correlated with a particular SNP.

a Clarithromycin isolates with MICs of 0.5 μg/mL (intermediate) (*n* = 2).

b “Product” designation in GenBank: AE000511.1.

**Figure 2:**
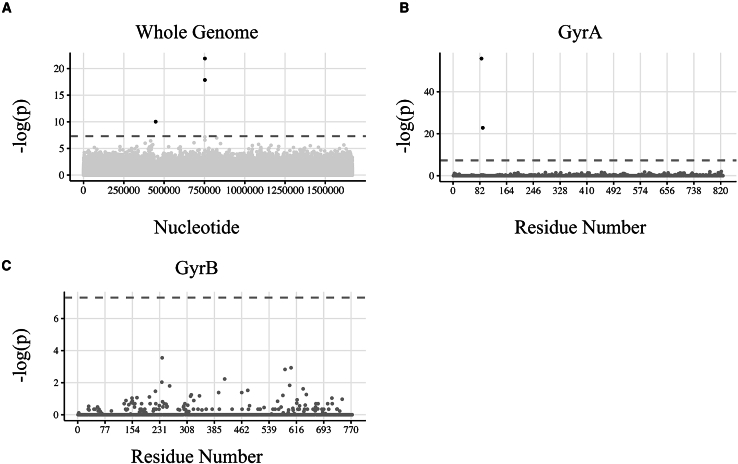
Manhattan plots illustrating results of a Fisher exact test for SNPs or missense mutations for *H. pylori* isolates tested for levofloxacin susceptibility (A) The -log(p) values of a Fisher exact test comparing the number of SNPs in phenotypically levofloxacin resistant (*n* = 240) versus susceptible (*n* = 290) isolates across the entire genome is plotted against the chromosome position of *H. pylori* ATCC 26695. (B) The -log(p) values of a Fisher exact test comparing missense mutations in levofloxacin resistant versus susceptible isolates across GyrA reveals enrichment of missense mutations in N87 and D91. (C) The -log(p) values of a Fisher exact test comparing the number of missense mutations in levofloxacin resistant versus susceptible isolates across GyrB did not reach statistical significance (see additional data in Figure S2). A dashed gray line shows a significance cutoff value of *p* ≤ 5e-8; significant nucleotides or amino acid residues are shown in black.

Only SNPs in *HP_r01* and *gyrA* were enriched in levofloxacin resistant isolates (Figure 2A; Table 2). Although SNPs in *HP_r01* were enriched in levofloxacin-resistant isolates, 130/240 levofloxacin-resistant isolates were co-resistant to clarithromycin (see subsequent results). When these 130 clarithromycin-resistant isolates were removed and the analysis repeated, there was no enrichment for mutations in the 23S rRNA gene among levofloxacin-resistant isolates. In a targeted analysis, missense mutations at residues 87 and 91 (Figure 2B) of GyrA were enriched in levofloxacin-resistant isolates. Interestingly, 33 phenotypically levofloxacin-resistant isolates demonstrated “wild-type” sequences at residues 87 and 91 (either asparagine or threonine; Tables S3 and S4). These isolates were not enriched for missense mutations at other locations along GyrA compared to levofloxacin-susceptible isolates (Figure S2A). Missense mutations were not enriched at a particular residue in GyrB for the entire dataset (Figure 2C). Among the 33 phenotypically levofloxacin-resistant isolates displaying genotypic wild-type GyrA at residues 87 and 91, there were no enriched missense mutations in GyrB (Figure S2B).

Amoxicillin-resistant isolates were enriched for SNPs in 11 genes (Table 2), including *fabD* (*HP_0090*), *cag17* (*HP_0538*), and *ung* (*HP_1347*). Several SNPs correlated with resistance phenotypes had missense mutations that did not correlate with the corresponding amino acid residue in these genes (Figure S3). For example, a SNP in *cdsA* (*HP_0215*) correlated with amoxicillin resistance, but the corresponding missense mutation was not correlated with amoxicillin resistance. This may be due to several SNPs being silent mutations. However, several SNPs and their corresponding missense mutations, such as in TrpS (HP_1253) and Ung (HP_1347), were correlated with amoxicillin resistance. Of the genes in which the SNP and the corresponding amino acid mutation were both significantly enriched in amoxicillin-resistant isolates, *cag* pathogenicity island protein CagN (*cag17*) is associated with type 4 secretion systems and possibly translocation of proinflammatory CagA,^63^ although the biological function and clinical significance of *cag17* specifically is unclear,^64^^,^^65^^,^^66^ and *HP_1347* corresponds to an uncharacterized uracil-DNA glycosylase (*ung*) homolog.^67^ Further, based on reports in the literature that amoxicillin resistance is correlated with mutations in PBPs, and in particular *pbp1a*,^30^^,^^32^^,^^68^^,^^69^^,^^70^^,^^71^^,^^72^^,^^73^^,^^74^ each penicillin binding protein was individually analyzed for missense mutations enriched in resistant isolates. There was no association between any of the PBPs and a particular missense mutation in the resistant versus susceptible isolates. For Pbp1A, 517 isolates had the wild-type sequence S414, with three susceptible isolates having S414N and no resistant isolates having missense mutations in S414 to either Arg or Asn, in contrast to previously published sequences of amoxicillin-resistant isolates.^75^ Similarly, resistance to amoxicillin has been ascribed to impaired uptake through decreased membrane permeability, which has been attributed to the outer membrane proteins (OMPs), HopB (HP_0913), HopC (HP_0912),^37^ HofH (HP_1167), and HefC (HP_0607),^76^ although other OMPs, such as HopT, HofC, and OMP31, were also upregulated in proteomic studies of clarithromycin-resistant *H. pylori* isolates.^77^ Here, there was no association between analyzed OMPs and missense mutations in resistant versus susceptible isolates.

Clarithromycin-resistant isolates were enriched for SNPs in the 23S rRNA gene (*HP_r01*), *vacA* (*HP_0887*), and the *cag16* (*HP_0537*) and *cag26* (*HP_0547*) pathogenicity islands (Figures S4A–S4E; Table 2). 260/302 clarithromycin-resistant isolates (86%) and 2/2 intermediate isolates harbored the well-characterized mutations A2147G, A2146G, or A2146C in the 23S rRNA gene (Table S5). The 42 phenotypic clarithromycin-resistant isolates that demonstrated wild-type adenosine nucleotides at bases 2146 and 2147 did not have enrichment of SNPs elsewhere in the genome (Figure S4F) or along the 23S rRNA gene (Figure S4G) compared to clarithromycin-susceptible isolates. Some enriched SNPs, such as those at nucleotide position 940652 in *HP_0887*, did not have an enriched missense mutation at the corresponding position (residue 746 in HP_0887), while others, such as those at nucleotides 940644 and 940645 in *HP_0887*, had both a SNP and a missense mutation enriched in resistant isolates.

Metronidazole resistance was correlated with SNPs in the 23S rRNA subunit (*HP_r01*; Table 2). However, 253/374 metronidazole-resistant isolates were co-resistant to clarithromycin, and 224/253 of these isolates had at least one of the known 23S rRNA gene mutations associated with clarithromycin resistance. Upon removing the 253 clarithromycin-resistant isolates and repeating the statistical analysis, there was no enrichment for mutations in the 23S rRNA subunit in the metronidazole-resistant isolates. Mutations in *frxA* and *fdxB* may play a role in metronidazole resistance in the presence of *rdxA*,^44^^,^^45^^,^^46^ although a specific mutation has not been observed,^47^^,^^48^^,^^50^^,^^51^^,^^52^ and resistant isolates were found to have wild-type *rdxA* sequences.^47^^,^^48^^,^^49^^,^^73^ In this study, there were no associations between metronidazole resistance and missense mutations in FrxA, FdxB, or RdxA (Figure S5). Several resistant isolates either matched the wild-type RdxA amino acid sequence or harbored mutations in RdxA not typically linked to metronidazole resistance.^73^

Correlation between rifampin phenotypic resistance and a particular SNP was not found. Furthermore, there were no associations between rifampin resistance and mutations in *rpoB* in this dataset (Figure S6).

Finally, while tetracycline has been associated with mutations in the binding site of the 16S rRNA sequence or efflux,^18^^,^^30^ the dataset did not include sufficient tetracycline-resistant isolates to pursue a detailed gene-by-gene analysis.

### Mutations in reported genes implicated in phenotypic resistance did not correlate with MIC values

To investigate genotype-phenotype correlation further, resistance genes were stratified by minimum inhibitory concentration (MIC) value rather than binary susceptible/resistant categorization (Figure S7). To highlight mutations that may be implicated in resistance, isolates with the highest and lowest MIC values were also compared, instead of binary susceptible/resistant categorizations. When stratifying by MIC for amoxicillin, mutations in Pbp1A were broadly present across the protein (Figure S7A), without significant enrichment of a particular missense mutation between isolates with the lowest MIC of <0.008 μg/mL and those with the highest MIC of >0.25 μg/mL (Figure S7B). The largest percentage difference between the lowest and highest MIC was residue 594 and 561. However, although 42% more isolates with an MIC value of <0.008 μg/mL harbored a missense mutation at residue 594 and 27% more isolates with an MIC value of >0.25 μg/mL contained a missense mutation at residue 561, these did not meet criteria for statistical significance (Fisher exact test; *p* ≈ 1.2e-6).

Stratifying the isolates by clarithromycin MIC (MIC: ≤0.25 and >0.5 μg/mL) highlights mutations strikingly enriched in the isolates with MICs >0.5 μg/mL, although mutations are still distributed throughout the 23S rRNA gene (Figures S7C and S7D). Similarly, stratifying the isolates by levofloxacin MIC (MIC: ≤1.0 and >1.0 μg/mL) highlights mutations in GyrA enriched in isolates with MICs >1.0 μg/mL, with mutations broadly distributed throughout the protein (Figures S7E and S7F). The percent difference between isolates in the two groups with a missense mutation or SNP was 17% (2146) and 58% (2147) for the 23S rRNA gene (clarithromycin), and 31% (residue 87) and 27% (residue 91) for GyrA (levofloxacin).

RdxA analysis (metronidazole, Figures S7G and S7H) did not reveal mutations associated with isolates with the higher MICs of 128 and 256 μg/mL versus the lowest MIC value of 8 μg/mL. For RpoB (rifampin, Figures S7J and S7K), although the percent difference in the number of isolates with mutations for the high MIC group (>2.0 μg/mL) versus the number of mutations for isolates in the low MIC group (0.12 or 0.25 μg/mL) was highest at residues 755 (28%) and 953 (25%), the percent difference at these residues did not meet the criteria for statistical significance (Fisher exact test; *p* ≈ 1.2e-6). The dataset did not include sufficient tetracycline-resistant isolates to pursue a detailed analysis stratifying by MIC versus binary susceptible/resistant categorizations.

### Number of missense mutations in reported resistance-associated genes did not correlate with phenotypic resistance

Overall, there was no statistically significant difference between the number of SNPs/missense mutations observed between resistant and susceptible isolates for each of the antibiotics across the genome and across their respective resistance-associated gene. Across the genome, the mean number of mutations in resistant versus susceptible isolates for amoxicillin (*p* = 0.59), levofloxacin (*p* = 0.47), metronidazole (*p* = 0.61), tetracycline (*p* = 0.98), and rifampin (*p* = 0.99) did not correlate with a resistance phenotype, while the number of mutations in clarithromycin resistant versus susceptible isolates did correlate with a resistant phenotype (*p* = 0.01). The average number of SNPs per isolate in the 23S rRNA gene did correlate with clarithromycin resistance (Welch’s *t* test: *p* = 6.8e-4; resistant: 4.8 SNPs; susceptible: 4.0 SNPs). However, there was no statistically significant correlation for the mean number of missense mutations per isolate and the respective resistance-associated gene for amoxicillin resistance and Pbp1A (Welch’s *t* test: *p* = 0.25; resistant: 46.0; susceptible: 48.0), levofloxacin resistance and GyrA (Welch’s *t* test: *p* = 0.50; resistant: 22.8; susceptible: 21.8), metronidazole resistance and RdxA (Welch’s *t* test: *p* = 0.21; resistant: 16.6; susceptible: 16.0), or rifampin resistance and RpoB (Welch’s *t* test *p* = 0.32; resistant: 202.0; susceptible: 198.6). The analysis was unable to be performed with tetracycline-resistant isolates and the 16S rRNA gene.

### Structural aberrations in reported genes implicated in resistance were not observed

Phenotypically resistant isolates may be enriched for mutation hotspots in a particular protein which may lead to subtle structural abnormalities, rather than one specific mutation affecting enzymatic function. The top 10 (by prevalence) observed mutations of the resistant and susceptible isolates (per antibiotic) in the presented dataset were mapped to the predicted protein structures of genes implicated in resistance.^78^^,^^79^^,^^80^

Mutations in both susceptible and resistant isolates were broadly distributed across reported resistance genes (Figure S8). Additionally, there was overlap between the most prevalent mutations in resistant and susceptible isolates for the respective antibiotics. Of the ten most common mutations in resistant and susceptible isolates, 9/10 mutations in GyrA (Figure S8A), 8/10 mutations in Pbp1A (Figure S8B), and 8/10 in RdxA (Figure S8C) were within the top ten most prevalent mutations in both resistant and susceptible isolates. Previous work by Tomb et al. indicates that RpoB in the *H. pylori* 26695 strain is a fusion of RpoB-RpoC,^81^ although this fusion is not necessary for viability.^82^ Both the RpoB and RpoC domains of RpoB (HP_1198) harbor mutations, and the mutations do not cluster preferentially in either of the RpoB or RpoC domains. 7/10 mutations were within the 10 most prevalent mutations in both resistant and susceptible isolates in RpoB-RpoC (Figures S8D and S8E), with similar numbers of mutations in the RpoB domain (resistant: 6/10; susceptible: 8/10) compared to the RpoC domain. Across each protein, some amino acid residues had >75% of both resistant and susceptible isolates containing that particular mutation.

## Discussion

*H. pylori*, whether antibiotic-resistant or -susceptible, has a highly diverse genome due to its high mutation rate driven by long-term co-evolution with human stomach microenvironments and resulting adaptation and diversification.^83^^,^^84^^,^^85^ Furthermore, *H. pylori* may exist as a quasispecies or a population of closely related, but genetically distinct variants within a single host. Combined with a high recombination rate,^86^ *H. pylori* is able to rapidly adapt to changing conditions. In the presented analysis, significant variability was observed between all isolates (regardless of phenotype) and the *H. pylori* 26695 reference strain (accession number: AE000511), which suggests that the reference strain may not fully reflect the sequence of commonly encountered clinical isolates.

Here, GWASs revealed a lack of correlation between phenotypic resistance and sequence for all tested antibiotics, aside from clarithromycin and levofloxacin. Aside from the well-characterized mutations associated with clarithromycin and levofloxacin resistance, other mutations in resistance-associated genes did not correlate with MICs and reported resistance-associated mutations for amoxicillin, metronidazole, rifampin, and tetracycline were not clearly associated with resistance. Similarly, the number of mutations within an isolates’ genome or within a resistance-associated gene did not correlate with phenotypic resistance. The most observed mutations did not reveal any structural clustering in resistance-associated genes that would otherwise obviously correspond to phenotypic resistance.

There are reports suggesting the presence of resistance-associated mutations.^73^^,^^74^^,^^75^^,^^87^^,^^88^ For example, S414R/N in Pbp1A,^75^ C19Y and T49K in RdxA,^73^ and multiple distinct amino acid changes across the conserved PBP-motifs (STGK_338–441_, SKN_402–404_, and KTG_555–557_) have been implicated in resistance,^36^^,^^70^ though these studies involved small collections of isolates. These mutations were observed inconsistently across both susceptible and resistant isolates in this study. Furthermore, several isolates that were resistant to levofloxacin (*n* = 30) or clarithromycin (*n* = 42) did not have any of the well-described mutations in *gyrA* or the 23S rRNA gene, respectively, highlighting current knowledge gaps. This has also been reported in a study by Lyu et al. reporting 6 out of 9 levofloxacin-resistant isolates having mutations at residue 87 or 91 of GyrA, with 6 levofloxacin-susceptible isolates also having mutations at residue 87 (D87K), and a number of study isolates additionally contained SNPs of unknown significance but with statistically significant enrichment in resistant isolates.^87^ However, in the Lyu et al. study, statistical correction for multiple testing was not implemented and a *p* value of ≤0.05 was used for the statistical significance threshold of a chi-squared analysis.

Given the genetic diversity of *H. pylori*, a publicly available and robust database of *H. pylori* sequences is essential for the development of molecular tools for AST prediction. While molecular tests are available to assess *H. pylori* resistance,^20^^,^^57^^,^^83^^,^^84^ none are currently FDA cleared. Based on the results presented, molecular prediction of clarithromycin and levofloxacin resistance or susceptibility is feasible, but it is less clear whether resistance or susceptibility to amoxicillin, metronidazole, and rifampin (and/or rifabutin) can be adequately captured based on existing knowledge. Further definition of underlying mechanisms of amoxicillin, metronidazole, and rifampin (and/or rifabutin) in *H. pylori* is needed.

### Limitations of the study

This study has several limitations. The first concerns the workflow between AST and WGS for studied isolates. 97.5% (753/773) of the isolates or 97.2% (515/530) of the bioinformatically analyzed isolates were sent to Mayo Clinic Laboratories for AST testing, with the rest of the isolates being from Mayo Clinic patients. For Mayo Clinic Laboratories-associated isolates, clinical history was unavailable, including patient location or birthplace (ordering client location was used as a proxy for geographic location) and patient treatment history (i.e., exposure to previous antibiotics). Furthermore, isolates were frozen, thawed, and regrown between AST and WGS. Cryopreservation may decrease mutational burden,^69^^,^^89^ though resistance markers have been detected directly from frozen gastric biopsies.^90^ Isolates may have been passed up to 3 times to obtain enough genetic material for short-read WGS, resulting in genetic variability from the original AST. Due to the fastidiousness of *H. pylori*, 243 isolates did not proceed to bioinformatic analysis (due to lack of growth, low DNA quality and/or bioinformatic quality control failure). It is possible that this subset of isolates had a unique feature which would not have been captured. For the analyzed 530 isolates, sufficient genetic material for paired long-read sequencing was not possible. As a consequence, all presented analysis is subject to the inherent limitations of short-read sequencing, such as difficulty with repetitive regions and complex genetic features.^91^ Such genetic features limit analysis of genes in which there are multiple copies, such as tetracycline and the 16S rRNA gene; this could be addressed in future studies with paired long-read sequencing.

Another limitation stems from technical limitations of phenotypic *H. pylori* AST. The activity of metronidazole is dependent on its reduction in an anaerobic environment,^92^ which is not used when testing *H. pylori* under microaerophilic conditions (and is not possible).^41^ False resistance to metronidazole may occur and has been documented for several aerotolerant anaerobes.^93^ Thus, metronidazole *H. pylori* MIC values may be artificially elevated and not correlate to clinical resistance. Similarly, whereas rifampin is typically used for phenotypic AST due to the existence of EUCAST (European Committee on Antimicrobial Susceptibility Testing) rifampin breakpoints and the lack of standardized methods for testing other rifamycins, rifabutin is more commonly used clinically.^4^^,^^55^^,^^94^ Importantly, phenotypic rifampin and rifabutin resistance are not always genotypically aligned.^54^^,^^55^^,^^56^ Furthermore, for all antibiotics, there can be variability in determined MICs. All isolates in this study underwent AST testing once; repeat testing may yield differing, but more precise MIC values.^95^^,^^96^^,^^97^ While MICs were measured per Clinical & Laboratory Standards Institute (CLSI) guidelines, isolates with inhibitory concentrations outside of the range of CLSI guidelines may have been artificially grouped together. The limited concentration ranges used to determine MICs (e.g., levofloxacin MICs ranging from 0.12 to 1.0 μg/mL) may have failed to capture mutations associated with MIC differences outside of the tested range.

Several challenges also exist for the *in silico* analysis and microbial GWAS,^98^ including filtering of low quality SNPs, accounting for phenotypic changes not associated with SNPs (deletions or insertions), accounting for lineage effects, and choosing an appropriate statistical cutoff for association (described in the STAR Methods section). In the case of *H. pylori*, a conservative approach was used, with a statistical threshold similar to human GWASs; however, there are various multiple testing corrections methods,^99^ with different strictness thresholds. Therefore, it is possible that mutations which confer low levels of non-susceptibility failed to meet the applied statistically significant threshold. Also, this analysis does not account for non-mutational mechanisms of resistance, including metabolic adaptation,^27^ upregulation of efflux pumps and specific OMPs,^77^^,^^100^ epigenetic effects,^101^^,^^102^ horizontal gene transfer,^103^^,^^104^ and homologous recombination.^67^^,^^105^^,^^106^

The population structure of *H. pylori* may also bias associations observed.^107^ *H. pylori* isolates cluster by patient populations, rather than geographic location.^108^ While the ordering provider’s geographic location was used as a proxy for isolate location, the patient’s *H. pylori* strain may instead reflect the geographic location of the patient when they acquired the infection. To minimize bias, a conservative *p* value threshold was used for GWASs and all associations validated with the corresponding amino acid mutation to minimize the risk of overcalling spurious associations. Similarly, experimental protein structures were not available for the structural mapping of the most frequent mutations. While protein predictions from AlphaFold have revolutionized structural biology, these predictions can deviate from experimental models on a global and local scale^109^^,^^110^ and predicting the impact of multiple, simultaneous amino acid mutations on protein structure is challenging.

Despite these limitations, results of this study show that *H. pylori* clinical isolates have significant genomic diversity, and that there is incomplete genotypic characterization of resistance mechanisms for amoxicillin, metronidazole, and rifampin.

## Resource availability

### Lead contact

Further information and requests for resources should be directed to and will be fulfilled by the lead contact, Robin Patel (patel.robin@mayo.com).

### Materials availability statement

This study did not generate new unique reagents.

### Data and code availability

• Genomic sequences and metadata for this paper are available in GenBank under the BioProject accession number GeneBank: PRJNA1242368. This accession should be used in citations to facilitate searching via Entrez.
• Code used for the analysis reported in this paper can be requested by reaching out to the lead contact.
• Any additional information required to reanalyze the data reported can be requested by reaching out to the lead contact.

## Acknowledgments

We thank the Bacteriology laboratory at Mayo Clinic, Rochester, Minnesota, for help with phenotypic susceptibility testing and, in particular, Nicolynn Cole and Angela Heitman. We acknowledge the Medical Genome Facility (Center of Individualized Medicine) at Mayo Clinic Rochester for help with the whole genome sequencing. Additionally, we thank Dr. Anika Iftekharuddin for critical reading and feedback on the manuscript. We gratefully acknowledge the following funding sources: Mayo Clinic Department of Laboratory Medicine and Pathology; Translational Research, Innovation, and Test Development Office; and the 10.13039/100014535Center for Individualized Medicine.

## Author contributions

Conceptualization, C.V., T.R., and R.P.; methodology, C.V., T.R., A.P.N., and R.P.; investigation, C.V., T.R., and S.J.; visualization, C.V. and T.R.; writing – original draft, C.V. and T.R.; writing – review & editing, C.V., T.R., S.J., A.N.S., A.P.N., and R.P.; funding acquisition, R.P.; resources, T.R., S.J., A.N.S., and R.P.; supervision, A.N.S., A.P.N., and R.P.

## Declaration of interests

Dr. R.P. reports grants from MicuRx Pharmaceuticals and bioMérieux. Dr. R.P. is a consultant to PhAST, Day Zero Diagnostics, DEEPULL Diagnostics, S.L., HealthTrackRx, bioMérieux, and CARB-X. In addition, Dr. R.P. has a patent on *Bordetella pertussis/parapertussis* PCR issued, a patent on a device/method for sonication, and a patent on an anti-biofilm substance issued. Dr. R.P. receives honoraria from Up-to-Date and the Infectious Diseases Board Review Course.

## STAR★Methods

### Key resources table

**Table:** 

REAGENT or RESOURCE	SOURCE	IDENTIFIER
**Bacterial and virus strains**		

*Helicobacter pylori*	ATCC	ATCC 43504

**Chemicals, peptides, and recombinant proteins**		

Becton Dickenson Brain Heart Infusion Broth	Cardinal Health	Cat#221813
Kendall Healthcare Precision™ Tissue Grinder System 50 mL, sterile, disposable	Cardinal Health	Cat#3500SA
Mueller-Hinton agar with 5% aged (≥2 weeks old) sheep blood	Difco-Fisher	Cat#241830
Amoxicillin	USP	Cat#1031503
Clarithromycin	USP	Cat#1134379
Levofloxacin	Millepore-Sigma	Cat#28266
Metronidazole	Millepore-Sigma	Cat#M3761
Rifampin	Millepore-Sigma	Cat#R3501
Tetracycline	Millepore-Sigma	Cat#T4062
Mitsubishi AnaeroPouch MicroAero gas generator	Thermo Scientific	Cat#R682005
Gas Tank: 10% CO2, balance mitrogen	Linde	Cat#BGNICD1C-K
Maxwell 16 Cell DNA purification kits	Promega	Cat#AS1020
AMPure XP beads	Beckman Coulter	Cat#NC9933872
NEBNext Ultra II DNA Library Prep Kit	New England Biolabs	Cat#E7645L
NEBNext multiplex oligos	New England Biolabs	Cat#E6440S
Illumina NovaSeq XP	Illumina	Cat#20043130
NovaSeq SP PE250 flow cell	Illumina	Cat#20028402
Acetonitrile, LC-MS Chromasolv (Sigma CAT# 34967) (HMMIS# 53150)	Honeywell Riedel-de Haën	60-046-514
70% Formic Acid	Sigma-Aldrich	94318-250ML
Water, LC-MS Chromasolv grade	Sigma-Aldrich	39253-1L-R
Trifluoroacetic acid	Sigma-Aldrich,	Cat#E7023-500,
Ethanol, 200 proof (absolute) ethyl alcohol, molecular grade 500 mL	Sigma-Aldrich	Cat#T6508
Biotyper® Sirius MALDI-TOF MS instruments with High Throughput (HT)	Bruker Scientific	
DNA suspension buffer	Teknova	Cat#T0223

**Deposited data**		

Whole genome sequences	This study	BioProject Accession: PRJNA1242368 Genebank ID: SAMN47587642 - SAMN48033501

**Software and algorithms**		

SKESA	Souvorov et al.^111^	https://github.com/ncbi/SKESA
CheckM (v1.1.0)	Parks et al.^112^	https://ecogenomics.github.io/CheckM/
Genome Taxonomy Database (GTDB) toolkit v.2.3.0	Chaumeil et al.^113^	https://gtdb.ecogenomic.org/
Bactopia’s Mashtree v3.0.0	Katz et al.^114^	https://bactopia.github.io/v3.0.0/bactopia-tools/mashtree/
ggtree	Yu et al.^115^	https://guangchuangyu.github.io/software/ggtree/
treeio	Wang et al.^116^	https://www.bioconductor.org/packages/release/bioc/html/treeio.html
R v4.3.1	R core Team, 2021^117^	https://posit.co/download/rstudio-desktop/
RStudio/2023.06.1+524	Rstudio Team, 2020^118^	https://posit.co/download/rstudio-desktop/
BWA v0.7.17	Li & Durbin, 2009	https://github.com/lh3/bwa
Samtools v1.18	Li et al.^119^	https://www.htslib.org/
BCFtools v1.9	Danecek et al.^120^	https://github.com/samtools/bcftools; https://samtools.github.io/bcftools/bcftools.html
Comprehensive Antibiotic Resistance Database (CARD) v3.2.5	Alcock et al.^121^	https://card.mcmaster.ca/
AlphaFold database	Varadi et al.^81^	https://alphafold.ebi.ac.uk/
UCSF ChimeraX	Goddard et al.^122^	https://www.cgl.ucsf.edu/chimerax/

### Experimental model and study participant details

#### Microbial isolates

773 *H. pylori* clinical isolates tested at Mayo Clinic (Rochester, MN) from 4/2021 to 5/2022 were studied. There was no selection of resistant or susceptible isolates; all isolates subjected to antimicrobial susceptibility testing (AST) which generated AST profiles during the study period were included. Of the 773 isolates, 97 were isolates with identification provided by a Mayo Clinic Laboratories (MCL) client, 670 were isolated at Mayo Clinic from specimens (667 stomach, 1 duodenum, 2 undefined) submitted for *H. pylori* culture and AST through MCL, and 6 were isolates recovered from gastric specimens of Mayo Clinic patients, identified as *H. pylori* and reflexed to AST. Geographically, 147, 323, 161, and 141 isolates were from Northeastern, Southern, Midwestern, and Western regions of the United States (Figure S1) as defined by the United States Centers for Disease Control and Prevention,^123^ with 1 isolate from Mexico. ATCC 43504 *H. pylori* was used as a control specimen for AST and WGS sequencing.

### Method details

#### Antimicrobial susceptibility testing

AST was performed using agar dilution as described by the Clinical and Laboratory Standards Institute (CLSI).^124^^,^^125^ Briefly, isolates were plated on Mueller-Hinton agar with 5% aged (≥2 weeks old) sheep blood and the following 2-fold dilutions: amoxicillin from 0.12 to 0.25 *μ*g/ml; clarithromycin from 0.25 to 0.5 *μ*g/ml; levofloxacin from 0.12 to 1.0 *μ*g/ml; metronidazole from 8 to 256 *μ*g/ml; rifampin from 0.12 to 2.0 *μ*g/ml; and tetracycline from 1.0 to 2.0 *μ*g/ml. Additional dilutions (0.008 and 0.06 *μ*g/ml) were included for quality control purposes for amoxicillin and tetracycline, respectively. Clarithromycin minimal inhibitory concentrations (MICs) were interpreted using CLSI breakpoints with ≤0.25, 0.5, and >0.5 *μ*g/ml classified as susceptible, intermediate, and resistant, respectively.^125^ European Committee on Antimicrobial Susceptibility Testing (EUCAST) breakpoints were used to interpret amoxicillin, levofloxacin, metronidazole, rifampin, and tetracycline results (Table S1).^126^ MIC breakpoints were ≤0.12 (S) and >0.12 (R) *μ*g/ml for amoxicillin, ≤1.0 (S) and >1.0 (R) *μ*g/ml for levofloxacin, ≤8 (S) and >8 (R) *μ*g/ml for metronidazole, ≤1 (S) and >1 (R) *μ*g/ml for rifampin, and ≤1 (S) and >1 (R) *μ*g/ml for tetracycline. After AST, isolates were placed in 5% defibrinated sheep blood and stored at -70°C until undergoing WGS.

#### Whole genome sequencing

The 773 isolates were retrieved from storage, thawed, plated on 5% sheep blood agar, and incubated under microaerophilic conditions (5% O_2_, 10% CO_2_, 85% N_2_) using the AnaeroPack System [Mitsubishi Gas Chemical Company, Inc.]) for 5-10 days. If needed, one to two additional passages were performed until enough growth was available to prepare a 3 McFarland standard suspension. DNA was extracted using Maxwell 16 Cell DNA Purification Kits (Promega). Extracted DNA was concentrated and cleaned using AMPure XP Beads (Beckman Coulter). 196 isolates did not proceed to WGS due to lack of growth, contamination, and/or low DNA quantity. Purified DNA concentrates from the remaining 577 isolates underwent library preparation using the NEBNext Ultra DNA Library Prep Kit (New England Biolabs) followed by sequencing on an Illumina NovaSeq XP using a NovaSeq SP PE250 flow cell.

### Quantification and statistical analysis

#### Genome assembly, indexing, &variant calling

Generated fastq files were assembled using SKESA (v2.4.0)^127^ with default parameters. Assembled whole genome sequences were submitted to DDBJ/ENA/Genbank under the BioProject accession PRJNA1242368. Genome quality was measured using CheckM (v1.1.0),^128^ with selection of genomes that displayed >90% completeness and <10% contamination and were classified as belonging to the genus *Helicobacter*. 47 generated genomes failed bioinformatic quality control, resulting in 530 genomes available for further analysis.

Species-level clustering was performed using the Genome Taxonomy Database (GTDB) toolkit.^111^ Genomic data was assembled in Bactopia,^112^ classified using the GTDB toolkit v.2.3.0, and the average nucleotide identity compared to isolates in GTDB (reference database release 214); isolates were clustered at the genus level according to the GTDB taxonomy category,^62^ with alphabetic suffixes of *H. pylori* subspecies reflecting polyphyletic genera according to the current GTDB reference tree or subdivided genera based on taxonomic rank normalization according to the current GTDB reference tree. Pairwise MASH distances were calculated using Bactopia’s Mashtree workflow (v3.0.0),^113^^,^^129^ and a dendrogram created using the ggtree^114^^,^^130^^,^^131^^,^^132^ and treeio^133^^,^^134^ libraries in R.

The 530 clinical isolates assembled from SKESA were indexed to the *H. pylori* 26695 reference strain [accession number: AE000511],^81^ using BWA v0.7.17 and samtools v1.18^128^ sam. Variant calling was completed in BCFtools (v1.9) using default parameters.^115^^,^^119^

#### Data filtering and statistical analysis

The dataset was analyzed for known common resistance-associated mutations using the publicly available Comprehensive Antibiotic Resistance Database v3.2.5 (CARD).^120^ While the CARD database detected mutations in the 23S rRNA gene (A2147G/A2143G, A2146G/A2142G, and A2146C/A2142C) in isolates with phenotypic clarithromycin resistance, which correspond to mutations associated with clarithromycin resistance for sequences, mutations of unknown significance were also identified at other positions, and a number of sequences associated with phenotypic resistance to other antibiotics either did not have predicted mutations, or had predicted mutations not well described in the literature (e.g., 470 isolates were flagged as having S494H and E572G mutations in *pbp2* but were susceptible to amoxicillin).

A multi-targeted approach (Figure S9) was therefore taken to identify genes with correlation to resistant phenotypes. First, an agnostic approach was executed in which the genome was searched for SNPs enriched in phenotypically resistant isolates. Genome-wide association studies (GWAS) are hypothesis-free and test thousands of variants across the genome to identify alleles associated with a phenotype.^98^ The entire genome was independently searched for each resistance phenotype for any variant at each nucleotide index (e.g., Figure 2A). Data filtering and analysis was completed in R studio (v2024.12.0.467).^121^^,^^135^ Data was further filtered to remove called mutations with quality scores of less than 200. Fisher exact tests were used to assess the correlation between phenotypic resistance and SNPs or amino acids mutations. Any SNP for which results of a Fisher exact test comparing the number of resistant versus susceptible isolates showed a p value below the cutoff was deemed to correlate with the respective resistance phenotype (Table 2).

Microbial GWAS are not as well characterized as human GWAS,^98^^,^^118^ and a p-value threshold for significance is not clearly established. A p value of ≤5e-8 (derived from Bonferroni correction for multiple testing in early human GWAS studies) was used to reflect a significant correlation between the mutation and the specific phenotypic resistance,^98^^,^^117^^,^^136^^,^^137^^,^^138^^,^^139^ although other studies do report a “suggestive association” threshold with p≤1e-5.^140^^,^^141^^,^^142^ The *H. pylori* 26695 genome is about 1.6 Mb and a Bonferroni correction for multiple testing (0.05 / 1.6e6) is ≈3e-8, suggesting the threshold of 5e-8 would sufficiently account for multiple testing artifacts. Quantile-quantile (QQ) plots of the data shown in Manhattan plots were calculated for all comparisons; a representative QQ plot shows deviation of p values observed for significant residues from the null hypothesis for GyrA and levofloxacin resistance (Figure S2C).

Then, a targeted approach was taken in which any genes containing enriched SNPs from the GWAS, as well as any genes implicated in resistance, were assessed for enriched missense mutations in phenotypically resistant isolates (e.g., Figures 2B and 2C).^143^ Missense mutations were manually encoded for each gene using standard codon tables.^144^ The following genes were curated for their implicated role in resistance: the 23S rRNA subunit (*HP_r01*) for its role in clarithromycin resistance;^145^^,^^146^ *gyrA (HP_0701*) and *gyrB (HP_0501*) for their role in levofloxacin resistance;^25^^,^^29^^,^^147^^,^^148^ *pbp1a (HP_0597)*, *pbp2* (*HP_1565)*, *ftsI* (*HP_1556*),^29^^,^^32^^,^^68^^,^^71^^,^^72^^,^^73^ *hopC* (*HP_0912*),^37^ *hofH* (*HP_1167*) and *hefC* (HP_0607)^76^ for their implicated role in amoxicillin resistance; *rdxA* (*HP_0954*), *frxA* (*HP_0642*), and *fdxB* (*HP_1508*; UniProt ID: O26038) for their implicated role in metronidazole resistance;^39^^,^^40^^,^^41^^,^^42^^,^^73^ and *rpoB* (*HP_1198*) for its implicated role in rifampin resistance.^27^^,^^54^^,^^149^^,^^150^ The isolate collection/data did not have sufficient statistical power to assess SNPs implicated in tetracycline resistance (n=7 resistant isolates). Additionally, for *gyrA* specifically, wild-type sequence for nucleotide 752,771 was considered to be an “A” corresponding to N87 (per *H. pylori* 26695 reference strain [accession number: AE000511]), or a “C” corresponding to T87 (per *H. pylori* strain J99).^151^ Clarithromycin isolates with MICs of 0.5 *μ*g/ml (intermediate) (n=2) were considered resistant for the GWAS.

For key resistance genes implicated in antibiotic resistance (*pbp1A* and amoxicillin, 23S rRNA gene and clarithromycin, *gyrA* and levofloxacin, *rdxA* and metronidazole, and *rpoB* and rifampin), several further analyses were completed. The number of SNPs or missense mutations as a correlative factor for antibiotic resistance was assessed by comparing the number of mutations in susceptible versus resistant isolates for each key resistance gene with a two-tailed Welch’s t-test. Additionally, the percent of isolates with mutations per residue per MIC for each key resistance gene was calculated with all isolates compared to the *H. pylori* 26695 reference strain [accession number: AE000511]. Then, the number of isolates with a particular mutation in the lowest MIC versus the highest MIC was compared by a Fisher exact test (significant if p≤5e-8) to explore if a particular mutation correlated with an increased MIC. As there were no additional associations observed in the comparison between isolates with clarithromycin MIC’s of ≤0.25 and >0.5 *μ*g/ml in the 23S rRNA gene, and the number of “intermediate” isolates was relatively small compared to the number of categorically resistant isolates (∼0.7%), additional GWAS (as described in Figure S4), for isolates with clarithromycin MIC’s of ≤0.25 or >0.5 *μ*g/ml (i.e., excluding “intermediate” isolates) was deemed unnecessary. For statistical power, antibiotics for which a specific MIC had <20 isolates were combined with the next highest or lowest MIC value to reach a threshold of at least 20 isolates per MIC value. For example, for metronidazole, there were only 4 isolates with an MIC value of 256 *μ*g/ml and therefore, these 4 isolates were combined with isolates with MIC values of 128 *μ*g/ml (n=98), resulting in a total of 102 isolates at ≥128 *μ*g/ml.

#### Structural analysis

Predicted protein structures for GyrA, Pbp1A, RdxA, RpoB, and RpoC were obtained from the AlphaFold Database.^78^^,^^79^^,^^80^ RpoB in *H. pylori* 26695 is predicted to be a fusion of RpoB-RpoC;^152^ however, only separate RpoB and RpoC structures were available in the AlphaFold Database. Model visualization and analyses were performed with the UCSF ChimeraX package.^153^^,^^154^ The ten most common mutations in resistant isolates and the ten most common mutations in susceptible isolates were mapped on predicted structures for each key resistance gene.
