# Supplementary material for: Whole genome sequence analysis of Helicobacter pylori isolates reveals incomplete characterization of antimicrobial resistance mechanisms
Source: iScience. 2025 Nov 17;28(12):114077. doi: 10.1016/j.isci.2025.114077 (PMC12718164; doi:10.1016/j.isci.2025.114077)
Supplement: Document S1. Figures S1–S9 and Tables S1–S5 [file mmc1.pdf]

iScience, Volume *28*

Supplemental information

Whole genome sequence analysis of *Helicobacter pylori* isolates reveals incomplete characterization of antimicrobial resistance mechanisms

Casey Vieni, Tanner Rothstein, Stephen Johnson, Audrey N. Schuetz, Andrew Norgan, and Robin Patel

Supplementary Table and Figure Legends

Table S1: Phenotypic characteristics of *Helicobacter pylori* study isolates, including those which failed downstream quality control.

Antibiotic	MIC (µg/ml) Breakpoint	Interpretive Category*	Isolates, N=773 (%)
Amoxicillin	≤0.12	S	698 (90.3)
	>0.12	R	75 (9.7)
Clarithromycin	≤0.25	S	331 (42.8)
	0.5	I	2 (0.3)
	>0.5	R	440 (56.9)
Levofloxacin	≤1	S	437 (56.5)
	>1	R	336 (43.5)
Metronidazole	≤8	S	244 (31.6)
	>8	R	529 (68.4)
Rifampin	≤1	S	563 (72.9)
	>1	R	209 (27.1)
Tetracycline	≤1	S	764 (98.3)
	>1	R	9 (1.32

MIC: minimum inhibitory concentration; S: Susceptible; I: Intermediate; R: Resistant; *Clarithromycin MICs were interpreted using CLSI breakpoints. Amoxicillin, levofloxacin, metronidazole, rifampin, and tetracycline MICs were interpreted using EUCAST breakpoints

Table S2: Antimicrobial drug resistance patterns of *Helicobacter pylori* isolates studied (based on phenotypic susceptibility testing).

Phenotype	Sequenced Isolates (N = 530)	Total Number of Isolates in Study (N = 773)
Resistant to ≥1 antibiotic	466	669
Susceptible to all tested antibiotics	64	104*
**Single Resistance**		
AMX	0	1
CLR	23	36
LVX	15	24
MET	67	90
RIF	19	23
TET	0	0
**2 Drug Resistance**		
AMX + CLR	1	2
AMX + LVX	0	0
AMX + MET	1	2
AMX + RIF	2	2
AMX + TET	0	0
CLR + LVX	13	20
CLR + MET	63	97
CLR + RIF	7	10
CLR + TET	0	0
LVX + MET	23	35
LVX + RIF	4	4
LVX + TET	0	0
MET + RIF	17	27
MET + TET	0	0
RIF + TET	1	1
**Triple Resistance**		
AMX + CLR + LVX	1	4
AMX + CLR + MET	2	4
AMX + CLR + RIF	1	2
AMX + CLR + TET	0	0
AMX + LVX + MET	0	1
AMX + LVX + RIF	0	0
AMX + LVX + TET	0	0
AMX + MET + RIF	3	4
AMX + MET + TET	0	0
AMX + RIF + TET	0	0
CLR + LVX + MET	94	122
CLR + LVX + RIF	3	7
CLR + LVX + TET	0	0
CLR + MET + RIF	14	25
CLR + MET + TET	0	0
CLR + RIF + TET	0	0
LVX + MET + RIF	8	11
LVX + MET + TET	0	0
LVX + RIF + TET	0	0
MET + RIF + TET	0	0
**4 Drug Resistance**		
AMX + CLR + LVX + MET	14	19
AMX + CLR + LVX + RIF	2	4
AMX + CLR + LVX + TET	0	0
AMX + CLR + MET + RIF	4	6
AMX + CLR + MET + TET	1	1
AMX + CLR + RIF + TET	0	0
AMX + LVX + MET + RIF	2	2
AMX + LVX + MET + TET	0	0
AMX + LVX + RIF + TET	0	0
AMX + MET + RIF + TET	0	0
CLR + LVX + MET + RIF	41	58
CLR + LVX + MET + TET	2	2
CLR + LVX + RIF + TET	0	0
CLR + MET + RIF + TET	0	0
LVX + MET + RIF + TET	0	0
**5 Drug Resistance**		
AMX + CLR + LVX + MET + RIF	15	18
AMX + CLR + LVX + MET + TET	0	0
AMX + CLR + LVX + RIF + TET	0	0
AMX + CLR + MTZ + RIF + TET	0	0
AMX + LVX + MTZ + RIF + TET	0	0
CLR + LVX + MET + RIF + TET	2	2
**6 Drug Resistance**		
AMX + CLR + LVX + MET + RIF + TET	1	3
*Rifampin results were unable to be obtained for one isolate, but could be tested against other antimicrobials		
AMX: Amoxicillin; CLR: Clarithromycin; LVX: Levofloxacin; MTX: Metronidazole; RIF: Rifampin; TET: Tetracycline		

Table S3: Isolate codons and amino acid sequences for residues 87 and 91 of GyrA in phenotypically levofloxacin-susceptible isolates.

**Phenotypically Susceptible Isolates (N=290)**				**GyrA Residue 91**				
				Wild Type	Mutant			
			**Amino Acid**	Aspartic Acid (D)	Glycine (G)	Tyrosine (Y)	Asparagine (N)	
		**Amino Acid**	**Codon**	GAT	GGT	TAT	AAT	Total
**GyrA Residue 87**	Wild Type	Asparagine (N)	AAC	140	3	0	0	238
			AAT	95	0	0	0	
		Threonine (T)	ACC	32	0	0	0	48
			ACT	15	0	0	1	
	Mutant	Lysine (K)	AAA	2	0	0	0	3
			AAG	1	0	0	0	
		Isoleucine (I)	ATC	1	0	0	0	1
			ATT	0	0	0	0	
		Tyrosine (Y)	TAC	0	0	0	0	0
			TAT	0	0	0	0	
			Total	286	3	0	1	282*

*No observed mutations in residue 87 and/or 91

Table S4: Isolate codons and amino acid sequences for residues 87 and 91 of GyrA in phenotypically levofloxacin-resistant isolates.

**Phenotypically Resistant Isolates (N=240)**				**GyrA Residue 91**				
				Wild Type	Mutant			
			**Amino Acid**	Aspartic Acid (D)	Glycine (G)	Tyrosine (Y)	Asparagine (N)	
		**Amino Acid**	**Codon**	GAT	GGT	TAT	AAT	Total
**GyrA Residue 87**	Wild Type	Asparagine (N)	AAC	14	11	6	13	85
			AAT	16	7	6	12	
		Threonine (T)	ACC	3	0	1	3	14
			ACT	0	0	1	6	
	Mutant	Lysine (K)	AAA	45	2	0	0	62
			AAG	15	0	0	0	
		Isoleucine (I)	ATC	48	0	1	2	77
			ATT	25	0	0	1	
		Tyrosine (Y)	TAC	0	1	0	0	2
			TAT	1	0	0	0	
			Total	167	21	15	37	33*

*No observed mutations in residue 87 and/or 91

Table S5: Isolate nucleotide sequences for nucleotide positions 2146 and 2147 in the 23S ribosomal RNA gene (*HP_r01*) of clarithromycin resistant and susceptible isolates.

	Phenotype	Sequence	A2147 / A2143 (Wild-type)	A2147G / A2143G	Total
Number of Isolates (N=530) with Mutations:	Phenotypically Susceptible (N = 226; MIC <0.5 µg/ml)	A2146 / A2143 (Wild-type)	226	0	226
		A2146G / A2142G	0	0	0
		A2146C / A2142C	0	0	0
	Phenotypically Intermediate (N = 2; MIC: 0.5 µg/ml)	A2146 / A2143 (Wild-type)	0	1	1
		A2146G / A2142G	1	0	1
		A2146C / A2142C	0	0	0
	Phenotypically Resistant (N = 302; MIC >0.5 µg/ml)	A2146 / A2143 (Wild-type)	42	206	248
		A2146G / A2142G	50	0	50
		A2146C / A2142C	4	0	4
			323	207	323

Figure S1: Geographic origins of isolates (N=773) tested at Mayo Clinic from 4/2021 to 5/2022 for *Helicobacter pylori* culture and/or antimicrobial susceptibility testing, colored by geographic region (Northeast, South, Midwest, West). 1 sample originating from Mexico is not shown.

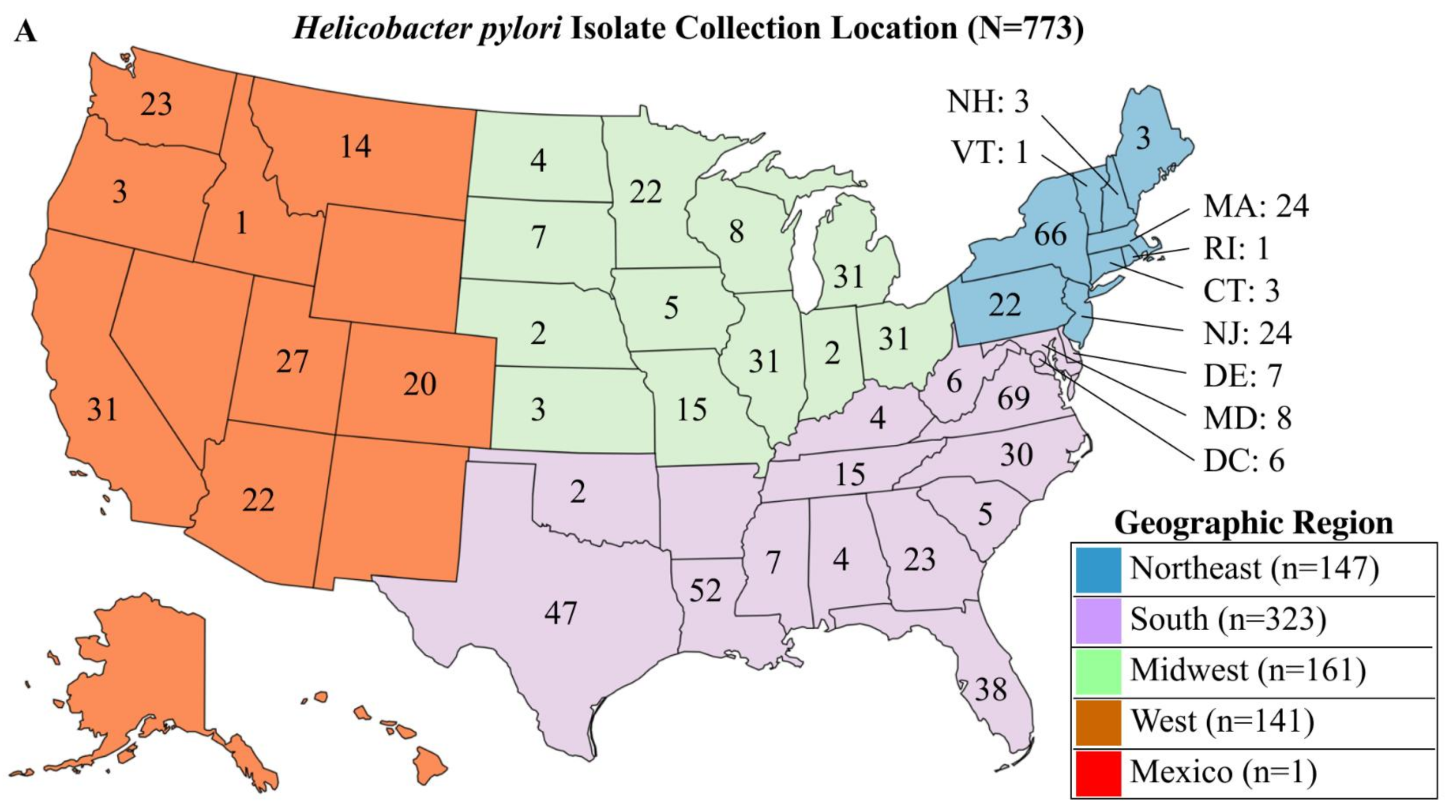

Figure S2: Manhattan plot and quantile-quantile (QQ) plot for isolates tested for levofloxacin susceptibility. (A) The -log(p) values of a Fisher exact test of a subset of levofloxacin resistant isolates with wild-type sequences in GyrA (N/T87 & D91; n=33) across the amino acid positions in (A) GyrA or (B) GyrB. A dashed gray line shows a significance threshold value of p≤5e-8 in panels A and B. (C) A QQ plot shows the distribution of observed p values for a Fisher exact test at each residue of GyrA compared to the theoretical distribution of p-values.

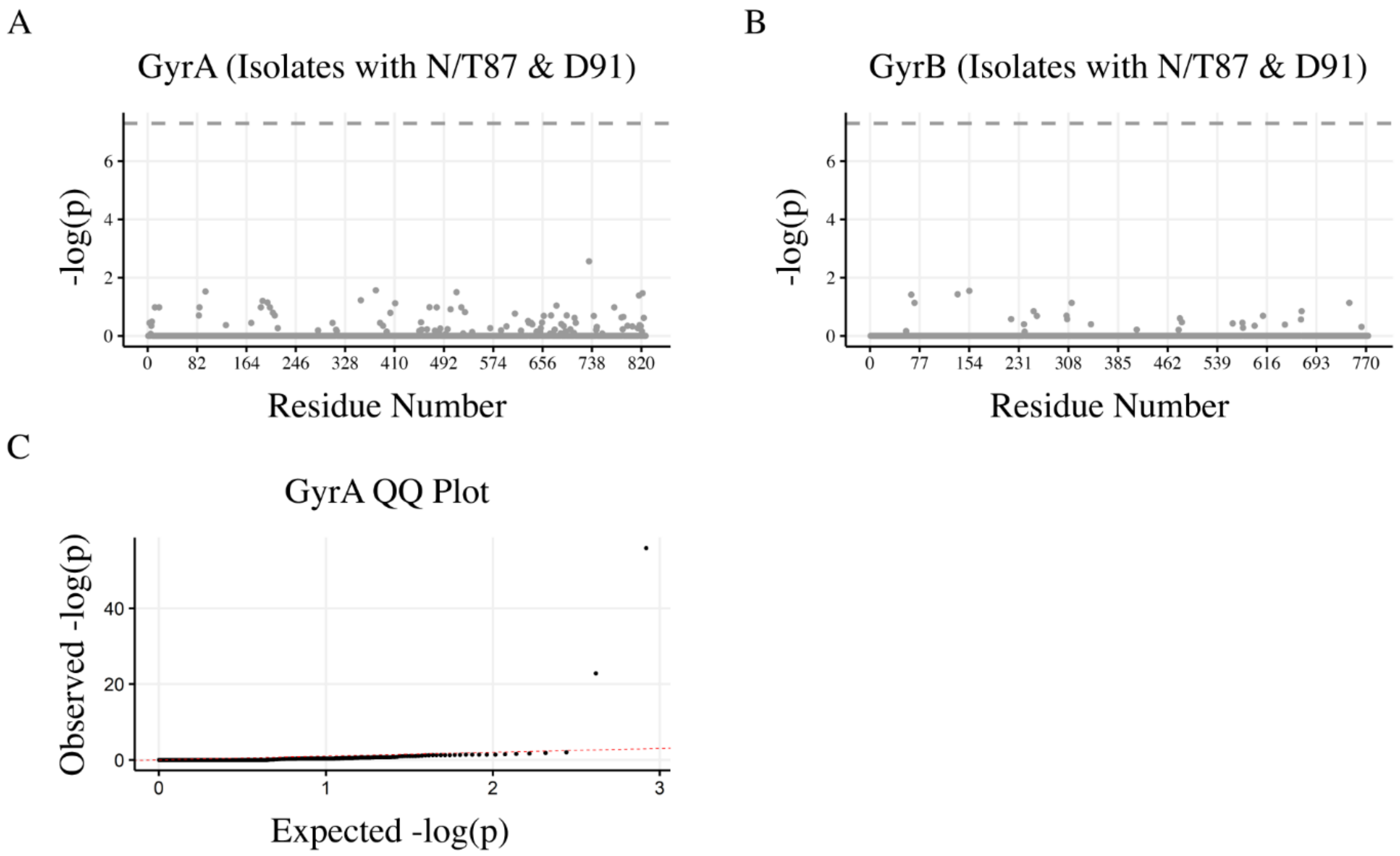

Figure S3: Manhattan plot illustrating results of a Fisher exact test comparing the number of single nucleotide polymorphisms (SNPs) or missense mutations for *Helicobacter pylori* isolates tested for amoxicillin susceptibility (susceptible: 480; resistant: 50). (A) The -log(p) values of a Fisher exact test of comparing the number of SNPs across the genome position of *H. pylori* ATCC 26695 in isolates tested for amoxicillin susceptibility. (B-L) The -log(p) values of a Fisher exact test comparing the number of missense mutations in isolates tested for amoxicillin susceptibility in the amino acid residues of (B) HP_0089, (C) HP_0090, (D) HP_0137, (E) HP_0215, (F) HP_0223, (G) HP_0522, (H) HP_0538, (I) HP_1186, (J) HP_1218, (K) HP_1253, (L) HP_1347, (M) Pbp1A, (N) Pbp2, (O) FtsI, (P) HefC, (Q) HopC, and (R) HofH. A dashed gray line shows a significance threshold value of p≤5e-8; significant nucleotides or amino acid residues are highlighted in black.

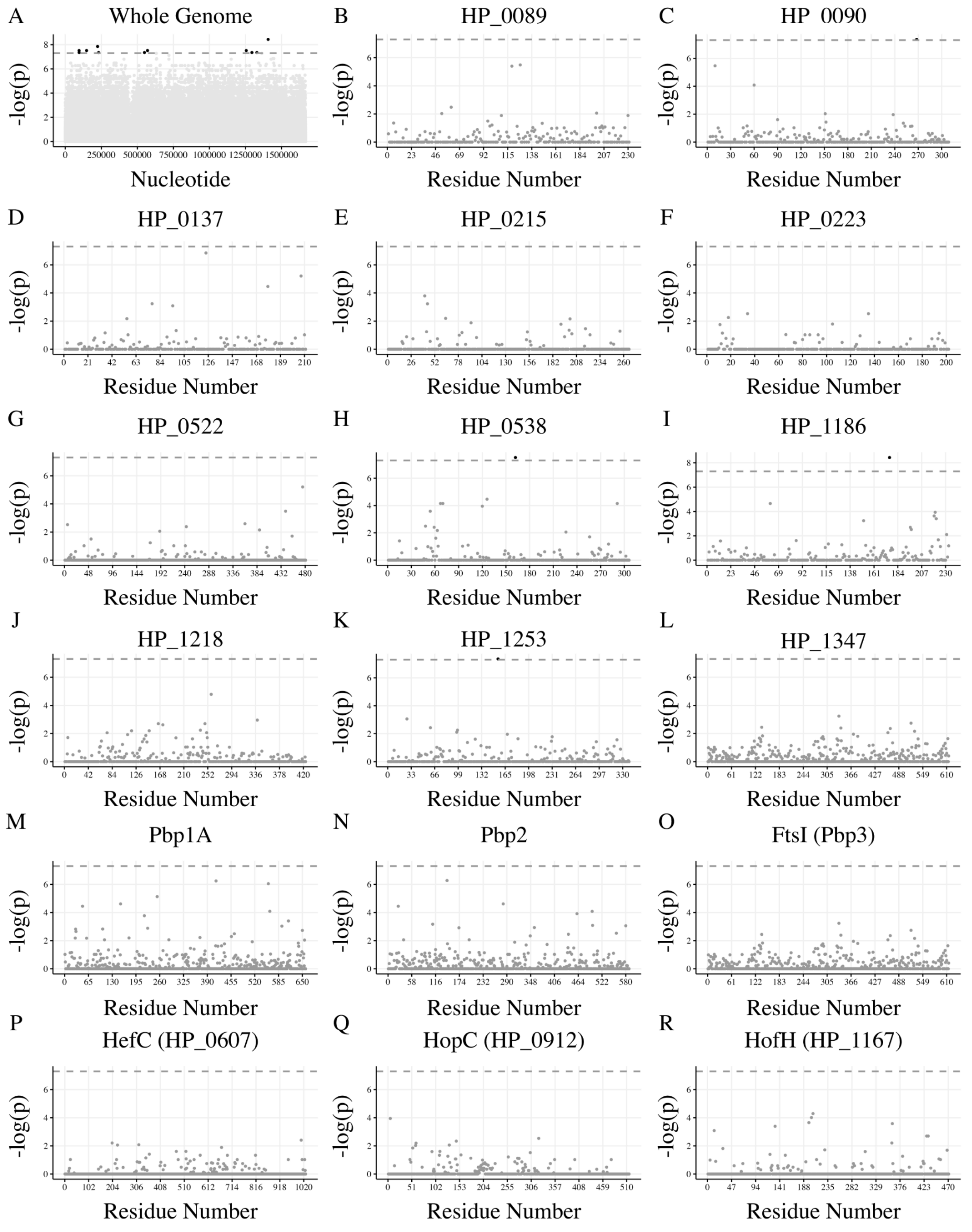

Figure S4: Manhattan plot illustrating the results of a Fisher exact test for single nucleotide polymorphisms (SNPs) or missense mutations for *Helicobacter pylori* isolates tested for clarithromycin susceptibility (susceptible: 226; intermediate & resistant: 304). (A) The -log(p) values of a Fisher exact test comparing the number of SNPs in isolates tested for clarithromycin susceptibility across the (A) genome position of *H. pylori* ATCC 26695 and (B) *HP_r01* (23S rRNA). SNPs corresponding to A2146 (2142) and A2147 (2143) were enriched in clarithromycin-resistant isolates. (C-E) The -log(p) values of a Fisher exact test comparing the number of missense mutations in isolates tested for clarithromycin susceptibility in the amino acid residues of (C) HP_0537, (D) HP_0547, and (E) HP_0887. (F-G) The -log(p) values of a Fisher exact test comparing the number of SNPs for a subset of clarithromycin resistant isolates with A2146 and A2147 in the 23S rRNA (*HP_r01*) gene (n=42) versus clarithromycin susceptible isolates plotted against nucleotide sequence (F) across the *H. pylori* genome and with (G) a focused view of the 23S rRNA gene. A dashed gray line shows the significance threshold value of p≤5e-8; significant nucleotides or amino acid residues are highlighted in black.

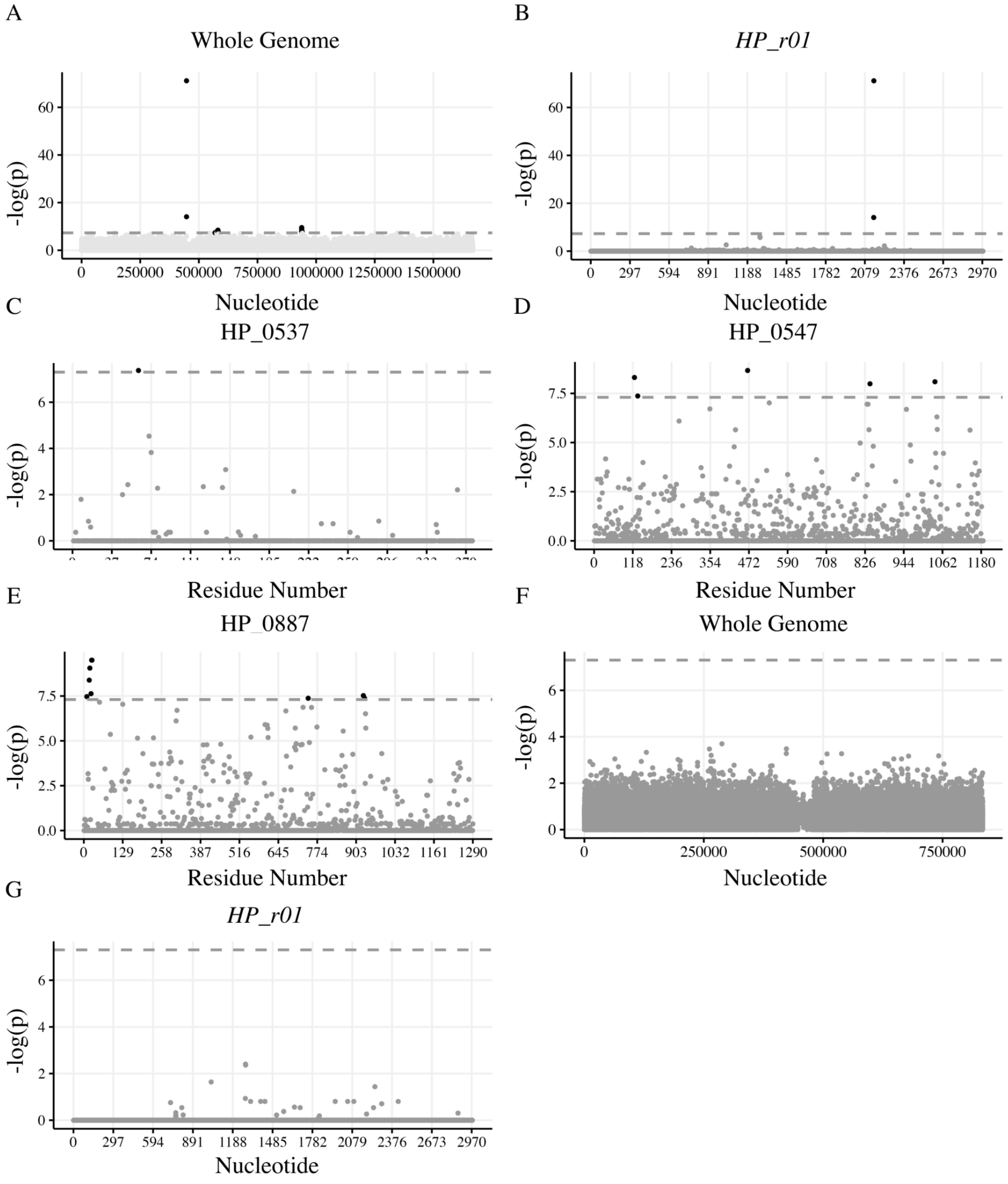

Figure S5: Manhattan plot illustrating results of a Fisher exact test comparing the number of single nucleotide polymorphisms (SNPs) or missense mutations for *Helicobacter pylori* isolates tested for metronidazole susceptibility (susceptible: 156; resistant: 374). (A) The -log(p) values of a Fisher exact test of comparing the number of SNPs across the genome position of *H. pylori* ATCC 26695 in isolates tested for metronidazole susceptibility. (B-D) The -log(p) values of a Fisher exact test comparing the number of missense mutations in isolates tested for metronidazole susceptibility across the amino acid residues of (B) RdxA, (C) FrxA, and (D) FdxB). A dashed gray line shows a significance threshold value of p≤5e-8; significant nucleotides or amino acid residues are highlighted in black.

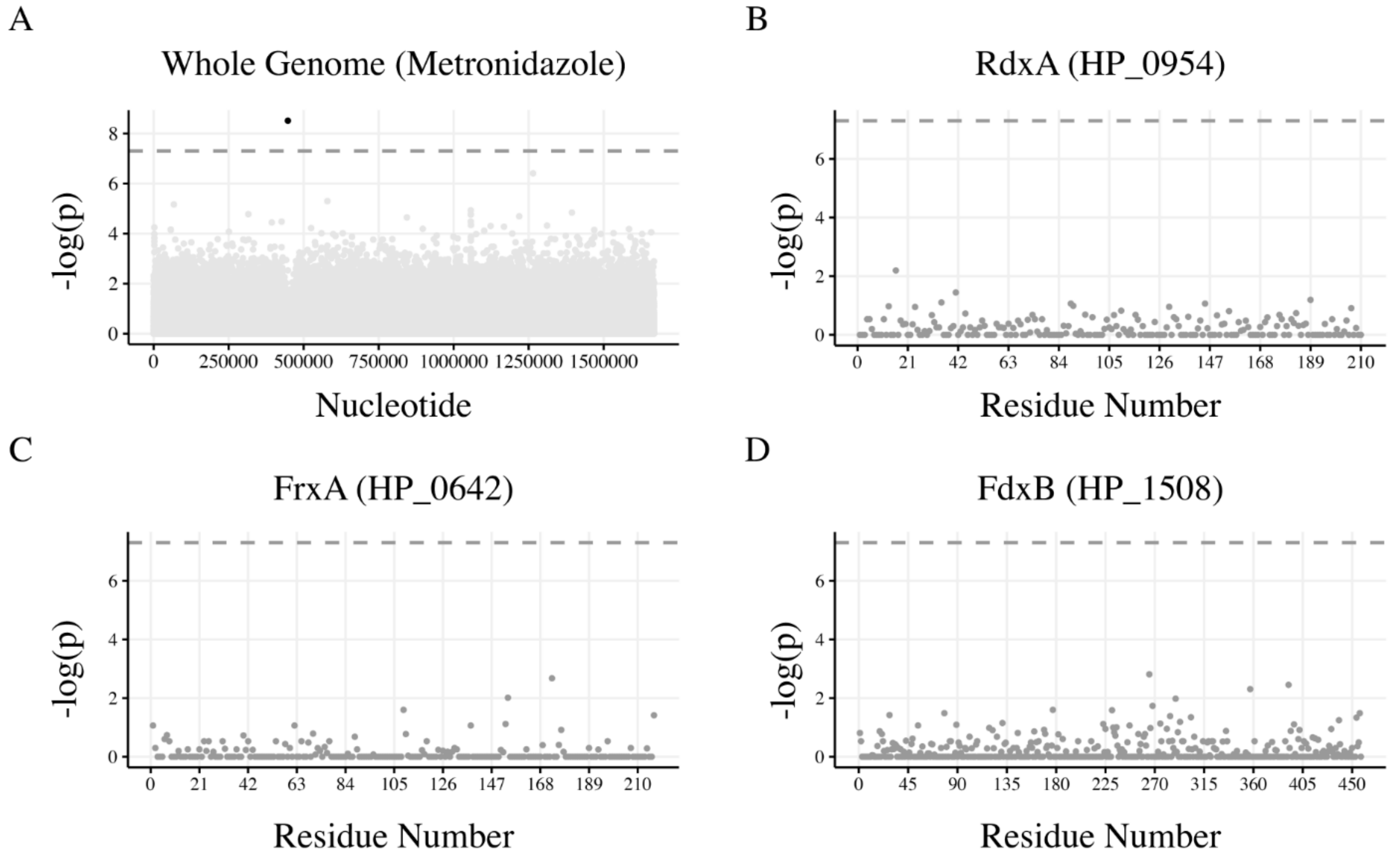

Figure S6: Manhattan plot illustrating the results of a Fisher exact test comparing the number of single nucleotide polymorphisms (SNPs) or missense mutations for *Helicobacter pylori* isolates tested for rifampin susceptibility (susceptible: 384; resistant: 146). (A) The -log(p) values of a Fisher exact test of comparing the number of SNPs across the genome position of *H. pylori* ATCC 26695 in isolates tested for rifampin susceptibility. (B) The -log(p) values of a Fisher exact test comparing the number of missense mutations in isolates tested for rifampin susceptibility across the amino acid residues of RpoB. A dashed gray line shows a significance threshold value of p≤5e-8; significant nucleotides or amino acid residues are highlighted in black.

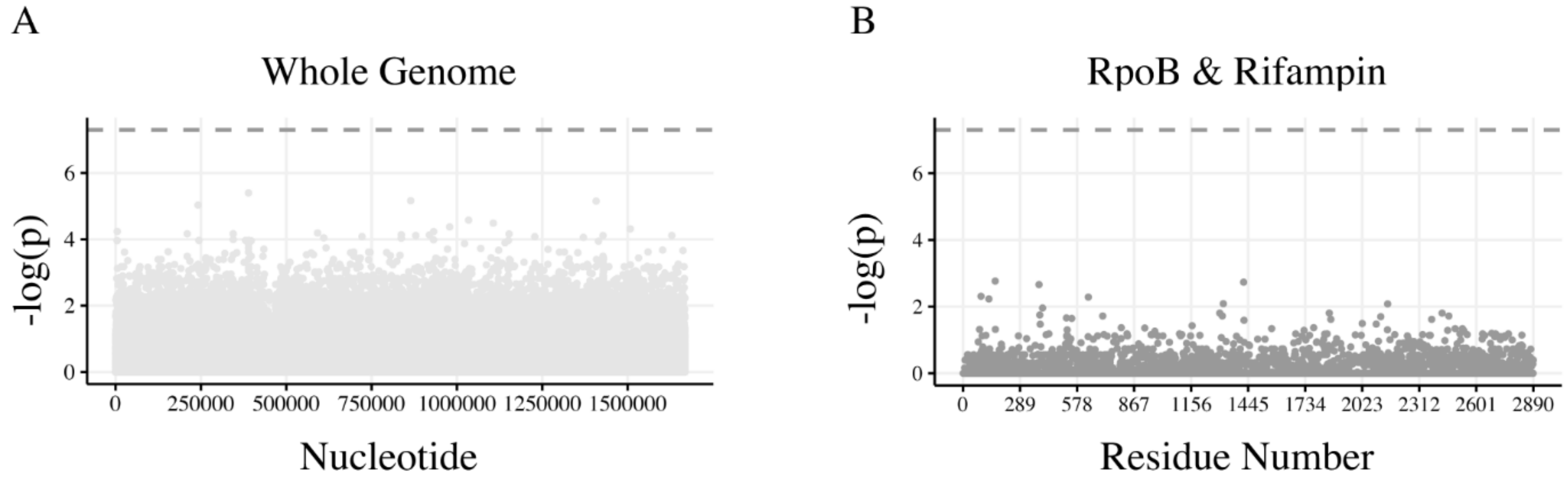

Figure S7: Comparison of the key resistance genes and their respective missense mutations stratified by minimum inhibitory concentration (MIC). (A) The percentage of isolates with a particular missense mutation is plotted against the amino acid residue number in Pbp1A. The isolates are stratified by amoxicillin MIC (MIC: <0.008, 0.12, 0.25, and >0.25 μg/ml). (B) The -log(p) values of a Fisher exact test comparing the number of susceptible and resistant isolates with a particular mutation show no statistically significant enrichment of a particular missense mutation in Pbp1A between the highest (>0.25 μg/ml) and lowest MIC (<0.008 μg/ml) tested for amoxicillin. (C) The percent of isolates with a particular single nucleotide polymorphism (SNP) is plotted against the location along the 23S rRNA subunit with the isolates stratified by their clarithromycin MIC (MIC: ≤0.25 and >0.5 μg/ml). (D) The -log(p) values of a Fisher exact test comparing the number of susceptible and resistant isolates with a particular mutation show statistically significant enrichment of SNPs at A2146 and A2147 in the 23S rRNA between the highest (>0.5 μg/ml) and lowest MIC (≤0.25 μg/ml) tested for clarithromycin. Isolates with a MIC corresponding to an intermediate interpretive category for clarithromycin were removed from this analysis. (E) The percentage of isolates with a particular missense mutation is plotted against the amino acid residue number in GyrA. The isolates are stratified by the levofloxacin MIC (MIC: ≤0.25, 0.5, 1.0, and >1.0 μg/ml). (F) The -log(p) values of a Fisher exact test comparing the number of susceptible and resistant isolates with a particular missense mutation show statistically significant enrichment for mutations in N87 and D91 of GyrA between the highest (>1.0 μg/ml g/ml) and lowest MIC (≤0.25 μg/ml) tested for levofloxacin susceptibility. (G) The percentage of isolates with a particular missense mutation is plotted against the amino acid residue number in RdxA. The isolates are stratified by the metronidazole MIC (MIC: 8, 16, 32, 64, 128, and 256 μg/ml). (H) The -log(p) values of a Fisher exact test comparing the number of susceptible and resistant isolates with a particular missense mutation show no statistically significant enrichment of a particular missense mutation in RdxA between the combined two highest MICs (MIC: 128 and 256 μg/ml) and lowest MIC (8 μg/ml) tested for metronidazole. (I) The percentage of isolates with a particular missense mutation is plotted against the amino acid residue number in RpoB. The isolates are stratified by the rifampin MIC (MIC: ≤0.12, 0.25, 0.5, 1.0, 2.0, and >2.0 μg/ml). (J) The -log(p) values of a Fisher exact test comparing the number of susceptible and resistant isolates with a particular missense mutation show no statistically significant enrichment of a particular missense mutation in RpoB between the highest MIC (>2.0 μg/ml) and the combined two lowest MICs (MIC: ≤0.12 and 0.25 μg/ml) for rifampin. Dashed gray lines are included in A, C, E, G, and I at 10, 25, and 50% of isolates to aid with visualization and a dashed gray line in B, D, F, H, and J shows a significance threshold value of p≤5e-8.

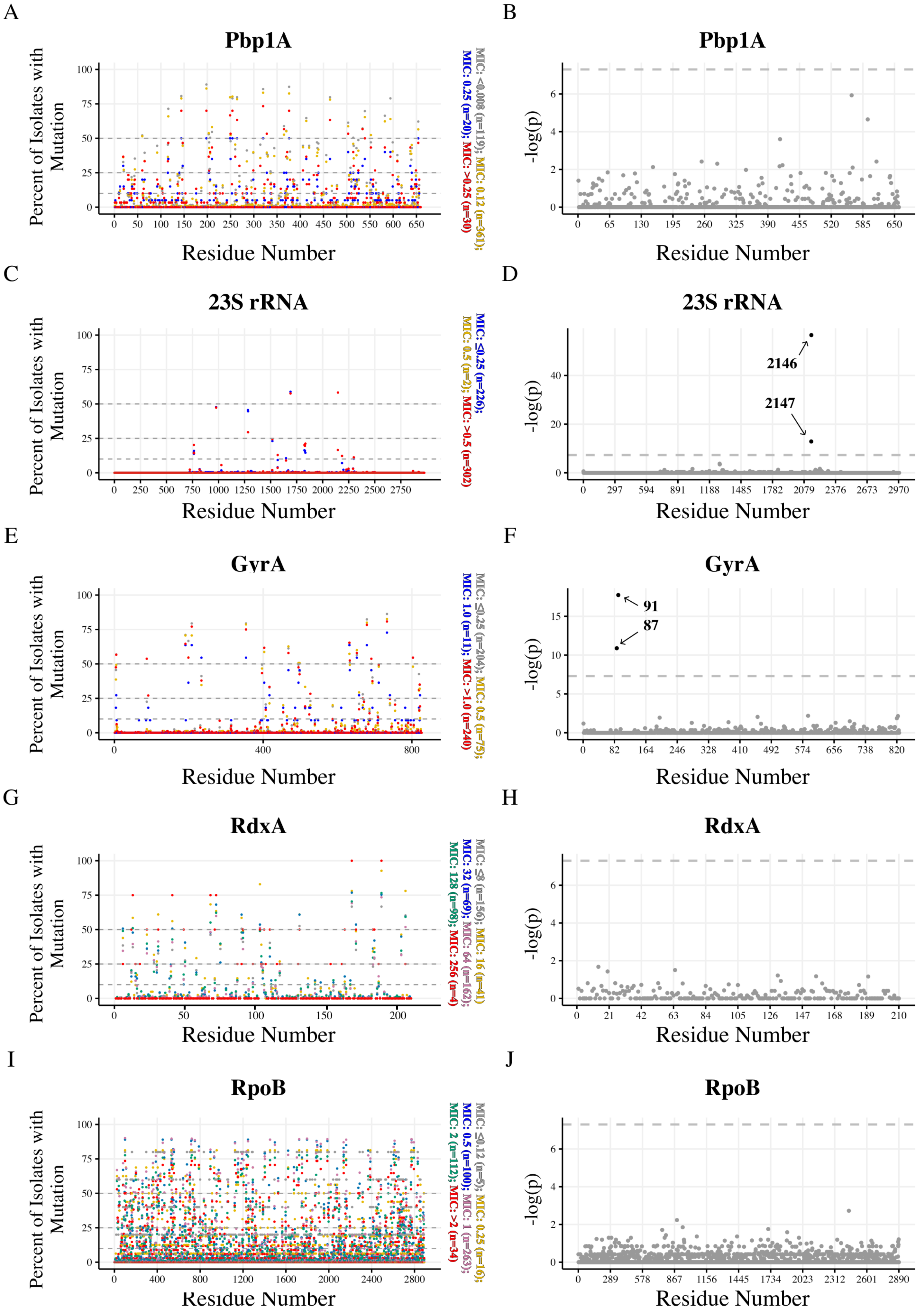

A
Pbp1A
Percent of Isolates with Mutation
Residue Number
MIC: ≤0.008 (n=119); MIC: 0.12 (n=361);
MIC: 0.25 (n=20); MIC: >0.25 (n=30)
B
Pbp1A
-log(p)
Residue Number
C
23S rRNA
Percent of Isolates with Mutation
Residue Number
MIC: ≤0.25 (n=226);
MIC: 0.5 (n=2); MIC: >0.5 (n=302)
D
23S rRNA
-log(p)
2146
2147
Residue Number
E
GyrA
Percent of Isolates with Mutation
Residue Number
MIC: ≤0.25 (n=204); MIC: 0.5 (n=75);
MIC: 1.0 (n=11); MIC: >1.0 (n=240)
F
GyrA
-log(p)
91
87
Residue Number
G
RdxA
Percent of Isolates with Mutation
Residue Number
MIC: ≤8 (n=150); MIC: 16 (n=41);
MIC: 32 (n=69); MIC: 64 (n=162);
MIC: 128 (n=98); MIC: 256 (n=4)
H
RdxA
-log(p)
Residue Number
I
RpoB
Percent of Isolates with Mutation
Residue Number
MIC: ≤0.12 (n=5); MIC: 0.25 (n=16);
MIC: 0.5 (n=100); MIC: 1 (n=263);
MIC: 2 (n=112); MIC: >2 (n=34)
J
RpoB
-log(p)
Residue Number

Figure S8: Structural comparison of the 10 most frequent mutations in resistant (red) and susceptible (green) isolates for predicted structures of key resistance genes for the antimicrobials studied. (A) GyrA (associated with levofloxacin resistance), (B) Pbp1A (associated with amoxicillin resistance), (C) RdxA (associated with metronidazole resistance), and the (D) RpoB domain and (E) RpoC domain of the RpoB-RpoC fusion protein (HP_1198; associated with rifampin resistance) for *H. pylori* 26695 reference strain [accession number: AE000511]. Mutations in yellow are shared between resistant and susceptible isolates, and mutations in blue correspond to key residues implicated in resistance.

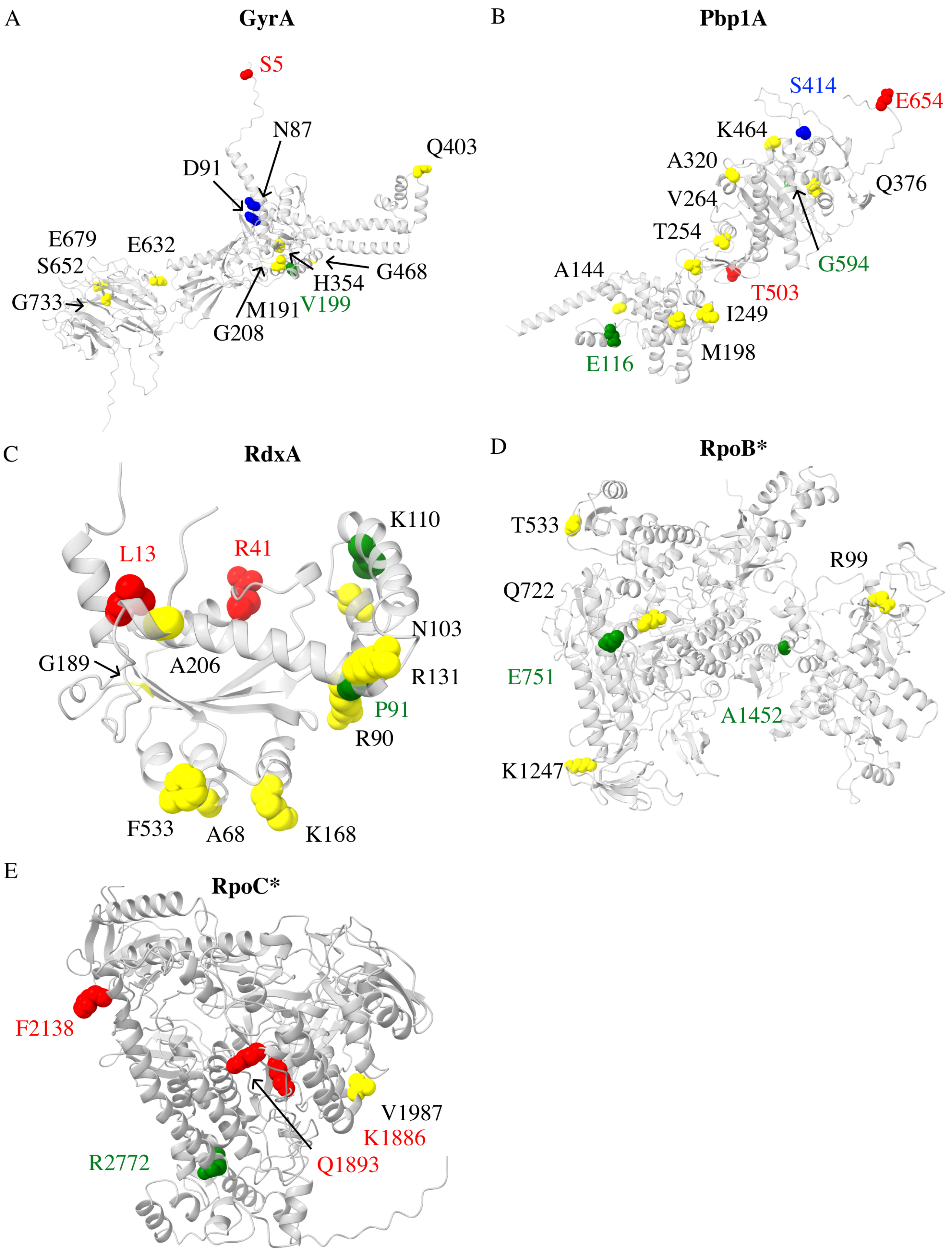

Figure S9: Data processing workflow for characterizing genes associated with phenotypic antimicrobial susceptibility results in *Helicobacter pylori*

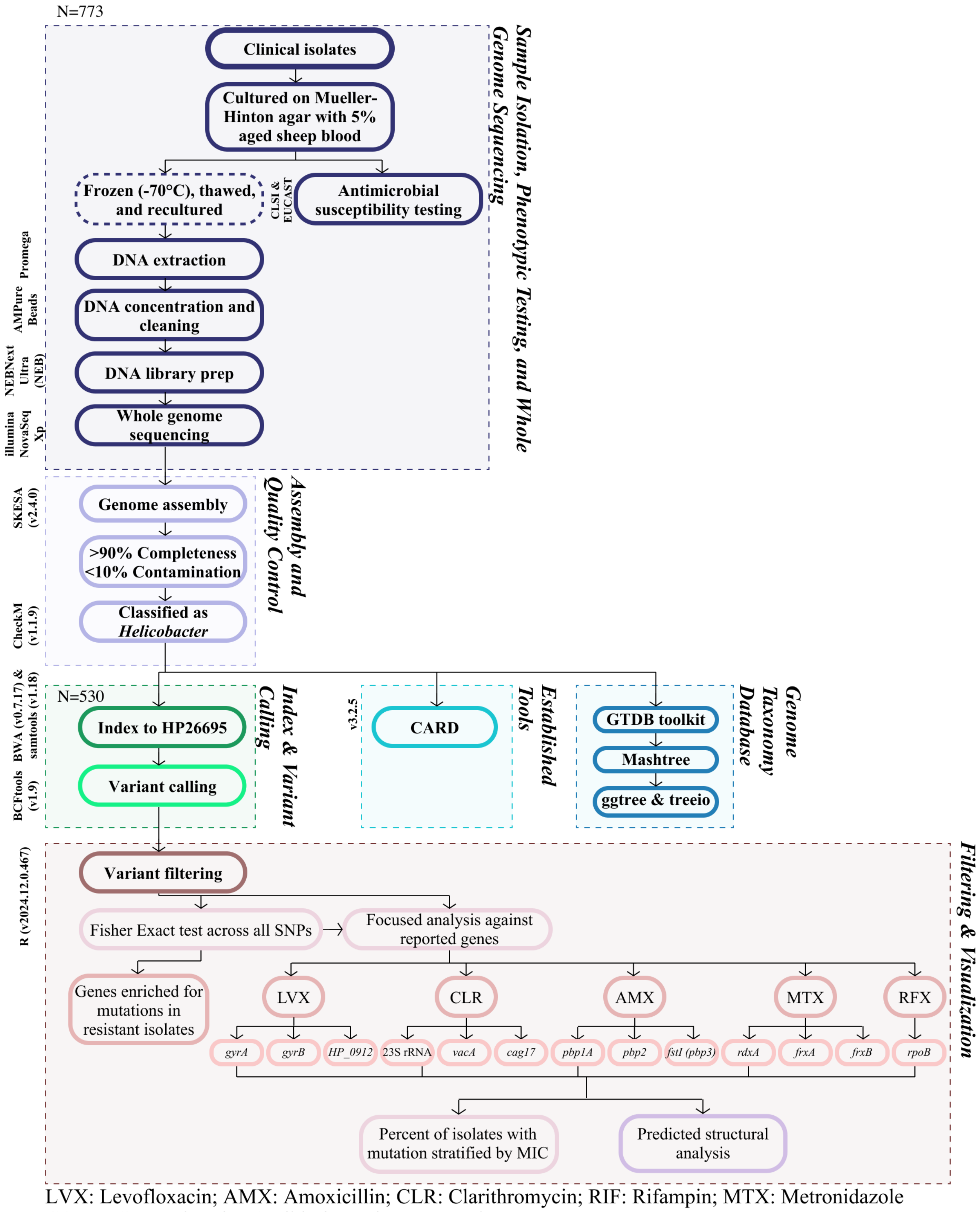

LVX: Levofloxacin; AMX: Amoxicillin; CLR: Clarithromycin; RIF: Rifampin; MTX: Metronidazole
CARD: Comprehensive Antibiotic Resistance Database
